# Divergent Evolution of the Transcriptional Network Controlled by Snf1-Interacting Protein Sip4 in Budding Yeasts

**DOI:** 10.1371/journal.pone.0139464

**Published:** 2015-10-06

**Authors:** Constance Mehlgarten, Jorrit-Jan Krijger, Ioana Lemnian, André Gohr, Lydia Kasper, Anne-Kathrin Diesing, Ivo Grosse, Karin D. Breunig

**Affiliations:** 1 Institute of Biology, Martin Luther University Halle-Wittenberg, Halle, Germany; 2 Institute of Computer Science, Martin Luther University Halle-Wittenberg, Halle, Germany; 3 German Centre for Integrative Biodiversity Research (iDiv) Halle-Jena-Leipzig, Leipzig, Germany; University Paris South, FRANCE

## Abstract

Cellular responses to starvation are of ancient origin since nutrient limitation has always been a common challenge to the stability of living systems. Hence, signaling molecules involved in sensing or transducing information about limiting metabolites are highly conserved, whereas transcription factors and the genes they regulate have diverged. In eukaryotes the AMP-activated protein kinase (AMPK) functions as a central regulator of cellular energy homeostasis. The yeast AMPK ortholog SNF1 controls the transcriptional network that counteracts carbon starvation conditions by regulating a set of transcription factors. Among those Cat8 and Sip4 have overlapping DNA-binding specificity for so-called carbon source responsive elements and induce target genes upon SNF1 activation. To analyze the evolution of the Cat8-Sip4 controlled transcriptional network we have compared the response to carbon limitation of *Saccharomyces cerevisiae* to that of *Kluyveromyces lactis*. In high glucose, *S*. *cerevisiae* displays tumor cell-like aerobic fermentation and repression of respiration (Crabtree-positive) while *K*. *lactis* has a respiratory-fermentative life-style, respiration being regulated by oxygen availability (Crabtree-negative), which is typical for many yeasts and for differentiated higher cells. We demonstrate divergent evolution of the Cat8-Sip4 network and present evidence that a role of Sip4 in controlling anabolic metabolism has been lost in the *Saccharomyces* lineag*e*. We find that in *K*. *lactis*, but not in *S*. *cerevisiae*, the Sip4 protein plays an essential role in C2 carbon assimilation including induction of the glyoxylate cycle and the carnitine shuttle genes. Induction of *KlSIP4* gene expression by KlCat8 is essential under these growth conditions and a primary function of KlCat8. Both KlCat8 and KlSip4 are involved in the regulation of lactose metabolism in *K*. *lactis*. In chromatin-immunoprecipitation experiments we demonstrate binding of both, KlSip4 and KlCat8, to selected CSREs and provide evidence that KlSip4 counteracts KlCat8-mediated transcription activation by competing for binding to some but not all CSREs. The finding that the hierarchical relationship of these transcription factors differs between *K*. *lactis* and *S*. *cerevisiae* and that the sets of target genes have diverged contributes to explaining the phenotypic differences in metabolic life-style.

## Introduction

Cell proliferation as well as survival of non-growing cells requires that anabolic (assimilation) and catabolic (dissimilation) metabolism are carefully balanced to meet the cellular demand for nutrients and energy. Despite the evolutionary divergences of regulators that assure cellular homeostasis, a set of protein kinases involved in metabolic regulation is highly conserved in eukaryotic cells, indicating an ancient common origin of metabolic control. One of those protein kinases is the adenosine monophosphate-activated protein kinase (AMPK), a central sensor of cellular energy status in yeast, plants and animals [1–4]. AMPK is allosterically regulated by phosphorylation via upstream kinases and the binding of adenosine phosphates. It affects glucose and insulin signaling in mammalian cells and has many roles in human disease [5,6]. Activation of AMPK leads to down-regulation of energy-consuming processes, like biosynthetic reactions, and to up-regulation of metabolic reactions providing energy [3]. The yeast homolog of AMPK, the Snf1 kinase complex (SNF1), is structurally highly related and regulated in a similar way. Mammalian upstream kinases can activate yeast SNF1 and *vice versa* [7–9]. Hence, studies in yeast have provided important insight into the molecular mechanisms by which the AMPK/SNF1 complex is regulated. These studies have also provided evidence that the appearance of the AMPK system was a very early event in evolution of eukaryotes and that the ancestral role of AMPK was in the response to starvation for a carbon source [10]. To unravel the transcriptional network controlled by AMPK and to trace back the complex networks found in higher eukaryotes to the ancient origin, characterization of the yeast SNF1-controlled network may also be instrumental.

In the early 20th century Otto Warburg discovered that metabolism in tumor cells is dominated by fermentation, which gives a low yield of ATP per glucose molecule consumed, whereas in differentiated cells energy production by the respiratory chain is prevailing unless oxygen is limiting (reviewed in [11]). This old observation has recently revived interest in the relationship between carbon metabolism and cancer and the balance between fermentation and respiration. The yeast *S*. *cerevisiae*, which is adapted to high sugar supply in its natural environment, performs aerobic fermentation like tumor cells and, like those, compensates the low energy yield by a high glycolytic flux. This leads to the so-called Crabtree effect: the formation of ethanol in the presence of oxygen [12,13]. Respiration and other mitochondrial activities are repressed by high glucose levels in the medium and need to be derepressed as glucose becomes scarce. The SNF1 complex plays an important role in this metabolic shift, which has been studied in great detail [9].

Most yeast species are Crabtree-negative. They regulate the carbon flux by oxygen availability and generate energy by respiration resembling differentiated animal cells in this respect [14]. Analysis of the SNF1-regulated transcriptional network in a yeast with such a respiro-fermentative life-style and comparison with a Crabtree-positive one may help to understand the ancient origin of the AMPK network. We have thus initiated a comparative analysis of SNF1-mediated gene regulation in the Crabtree-positive *S*. *cerevisiae* ("baker's yeast") and the distantly related Crabtree-negative yeast, *K*. *lactis* ("milk yeast") [15].

In both yeasts we study the reprogramming of gene expression that occurs when SNF1 is activated in response to carbon and/or energy limitation. Focusing on two SNF1-regulated transcription factors Cat8 and Sip4 [16,17] present in both *S*. *cerevisiae* as well as in *K*. *lactis* we identify and compare their target genes in the two species. We shift wild-type and mutant cells lacking Cat8 and/or Sip4 from a culture medium with the fermentable carbon source glucose to ethanol, which requires respiration to support growth, and analyze expression of genes that are known Cat8 targets in *S*. *cerevisiae* and their orthologs in *K*. *lactis*. Our goal is to find crucial differences between these species that help to explain their difference in life-style. In the long-run this may contribute to our understanding of what causes the metabolic differences in tumor vs. "normal" cells.

Cat8 and Sip4 belong to the large fungal-specific class of zinc cluster transcription factors [18]. They share a highly related DNA-binding domain with specificity for so-called carbon source responsive elements (CSREs) [19,20] but show little similarity in the rest of the protein. Cat8 has been characterized in *S*. *cerevisiae* (ScCat8) and *K*. *lactis* (KlCat8) as an activator of transcription [16,21]. It is activated by SNF1 via phosphorylation of a conserved serine residue (Ser-661 in KlCat8, Ser-562 in ScCat8) [22–24] and induces transcription of genes important for the metabolic shift that occurs upon glucose depletion [16,25–27]. Putative Cat8 orthologs have also been characterized in *Aspergillus nidulans (*FacB), *Aspergillus niger* (AcuB) and *Candida albicans* (CaCat8). Sip4 has only been studied in *S*. *cerevisiae*, so far. It has been identified in a screen for SNF1-interacting proteins and has been shown to be transcriptionally regulated by ScCat8 [17,28]. Since *Scsip4* single mutants have no apparent growth phenotype on any carbon source tested its biological role appears to be limited, at least in the presence of ScCat8. Despite the fact that more than 50 synthetic genetic interactions have been reported [http://www.yeastgenome.org/locus/S000003625/interaction] neither these epistatic relationships nor the potential ScSip4 target genes in the *S*. *cerevisiae* genome identified by chromatin immunoprecipitation [29] did reveal a specific GO process controlled by ScSip4.

*Sccat8* mutants are unable to grow on gluconeogenic carbon sources like ethanol or glycerol. In contrast, *K*. *lactis* requires KlCat8 for growth on ethanol but not on glycerol [21]. This indicates that the expression of the gluconeogenesis specific genes *FBP1* and/or *PCK1* (encoding fructose-1,6-bisphosphatase and phosphoenolpyruvate carboxykinase, respectively), which is essential for C3 carbon assimilation, is Cat8 independent in *K*. *lactis* whereas conversion of C2 carbon sources into pyruvate requires KlCat8.

Here we investigate the role of the gene *KLLA0F14322g* encoding the Sip4 homolog in *K*. *lactis* (KlSip4). We find that KlSip4 protein, in contrast to ScSip4, plays an important role downstream of KlCat8. It is required for mobilizing acetyl-CoA across intracellular membranes via the carnitine shuttle and for activation of the glyoxylate cycle. An additional copy of the *KlSIP4* gene can effectively suppress the deficiency of a *Klcat8*Δ mutant to grow on C2 carbon sources. This indicates that a primary role of KlCat8 is the activation of the *KlSIP4* gene. Apparently, a rewiring of the regulatory network under SNF1 control has occurred between the two yeasts where in *S*. *cerevisiae* at least some of the function of Sip4 has been adopted by Cat8. The resulting redundancy of Sip4 and Cat8 in *S*. *cerevisiae* might possibly be related to the divergence from a respiratory life style towards fermentation. We propose that the differences in the sets of Cat8 and Sip4 target genes might be linked to changes in subcellular acetyl-CoA partitioning.

## Results

### Overexpression *of KlSIP4* compensates for loss of *KlCAT8*

To identify KlCat8 target genes we exploited the inability of a *Klcat8*Δ mutant to grow on ethanol. The *Klcat8*Δ mutant strain was transformed with a *K*. *lactis* genomic library and plated on medium containing ethanol as the sole carbon source. Colonies selected in this way contained plasmids that fell in two classes: class I plasmids carried the *KlCAT8* gene, as expected, while class II plasmids, like pGP3, contained the open reading frame *KLLA0F14322g*, which shows high sequence similarity and synteny with *ScSIP4* and was annotated a*s KlSIP4* (http://www.ygob.ucd.ie; http://genolevures.org/klla.html). The fact that the *KlSIP4* plasmids suppressed the ethanol growth phenotype of the *Klcat8*Δ mutant indicated that elevated KlSip4 levels can compensate for KlCat8 function. Analogously, multicopy *ScSIP4* had been reported to suppress a *Sccat8* mutant [17].

**Fig 1:**
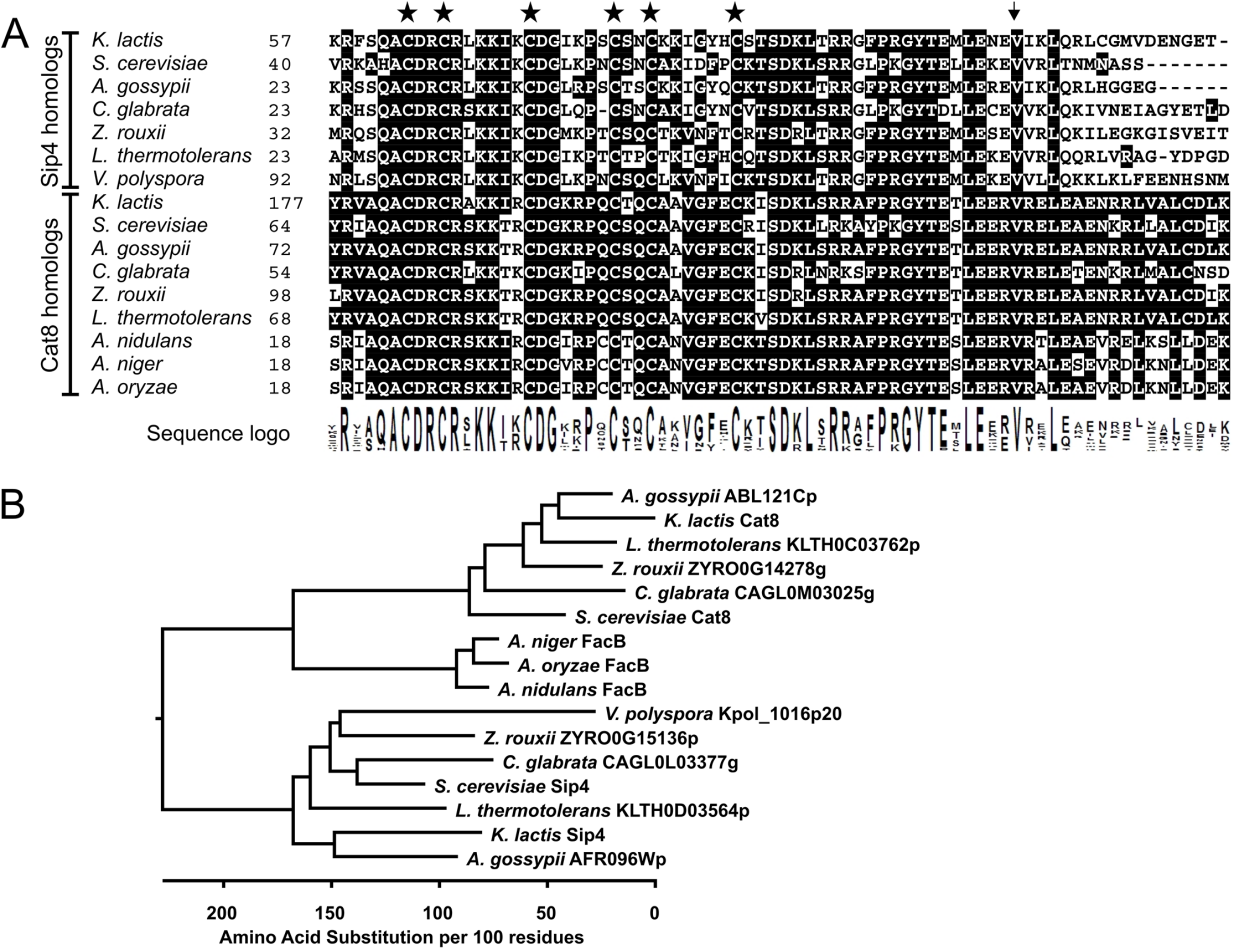
Sequence conservation in the Zn(II)_2_Cys_6_ fungal-type DNA-binding domain of Cat8 and Sip4 family members. (A) Multiple sequence alignment of fungal Zn(II)_2_Cys_6_ binuclear cluster motif, linker and coiled-coil regions of Sip4- and Cat8-like proteins derived from various fungal genomic sequences: *K*. *lactis*, *S*. *cerevisiae*, *A*. *gossypii*, *C*. *glabrata*, *Z*. *rouxii*, *L*. *thermotolerans*, *V*. *polyspora*, *A*. *nidulans*, *A*. *niger* and *A*. *oryzae*. Identical residues are shown in black boxes and the cysteine residues involved in the coordination of the Zn^2+^ atoms are marked with an asterisk. The arrow indicates a conserved valine residue following the point of sequence divergence in Cat8 vs. Sip4 as mentioned in the text. (B) Phylogenetic relationship based upon full-length amino acid sequences of Sip4 and Cat8 homologs in selected Ascomycota. The relationship is presented as a phylogram with branch lengths proportional to sequence deviation. Cat8 but no Sip4 homologs are found in *Aspergillus spp*..

It has been reported that ScSip4 and ScCat8 have a similar specificity for CSREs [30]. Consistently, their DNA-binding domains show high sequence conservation not only of the six cysteine residues involved in forming the Zn(II)_2_Cys_6_ binuclear cluster domain but also of the adjacent linker region that is involved in homodimerization and determination of DNA-protein geometry [31,32] (Fig 1A). Comparison of Cat8 and Sip4 protein sequences of homologs from related fungal species reveals that the similarity between the two protein families breaks within this linker region. A valine residue (arrow), which is highly conserved in both clades, is preceded by a basic amino acid (K or R) in Cat8 family members and by a glutamate (E) in the Sip4 family. From there, the sequences start to diverge, not only between Cat8 and Sip4 but also between Sip4 homologs, whereas the similarity between Cat8 homologs extends 17 amino acids further downstream. Sip4 homologs are found only in *Hemiascomycetes* where they cluster in a clade distinct from Cat8 whereas Cat8 homologs, like FacB, are also found in more distantly related genera like *Aspergillus spp*. (Fig 1B).

### KlSip4 is essential for C2 carbon assimilation

**Fig 2:**
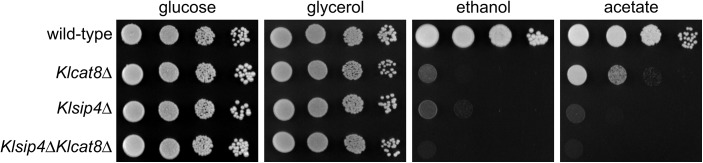
Influence of deletions in *KlCAT8* and *KlSIP4* on carbon utilization. *K*. *lactis* wild-type (JA6), *Klcat8*Δ (yIG8), *Klsip4*Δ (JA6/DS4) and *Klcat8*Δ*Klsip4*Δ (yIG8/DS4) strains were pregrown in YP medium with 2% glucose (see Material and Methods) and spotted in serial 10-fold dilutions on minimal plates with 2% of the indicated carbon sources. Plates were incubated at 30°C for 4 days.

To analyze whether KlSip4 is required for growth the *KlSIP4* gene was disrupted in the wild-type and *Klcat8*Δ mutant and the generated single and double mutants were phenotypically analyzed by spotting serial dilutions on different carbon sources (Fig 2, S1 Fig). Both strains lacking *KlSIP4* were greatly impaired for growth on ethanol and acetate but they grew like wild-type on glucose- and glycerol-media. This phenotype resembles that of the *Klcat8*Δ mutant [21] and contrasts with that of the *S*. *cerevisiae sip4*Δ mutant, for which no discernible effect on growth on any carbon source has been reported. Hence ScSip4 and KlSip4 have functionally diverged from their common ancestor.

Since KlSip4 is capable of complementing lack of KlCat8 when overproduced it apparently functions downstream of KlCat8. Strikingly, a single additional (HA-tagged) copy of *KlSIP4* (WT *KlSIP4 + KlSIP4-6HA*, see material and methods) did suppress the phenotype of the *Klcat8*Δ mutant and even partially suppressed the growth defect of a *Klsnf1*Δ mutant (S1 Fig). Hence, an important function of the KlSnf1 kinase appears to be the activation of the KlCat8-KlSip4 cascade upon growth on ethanol or acetate, and a crucial function of KlCat8 appears to be the activation of the *KlSIP4* gene.

### KlSip4 controls the expression of genes involved in C2 carbon assimilation and the glyoxylate cycle

**Fig 3:**
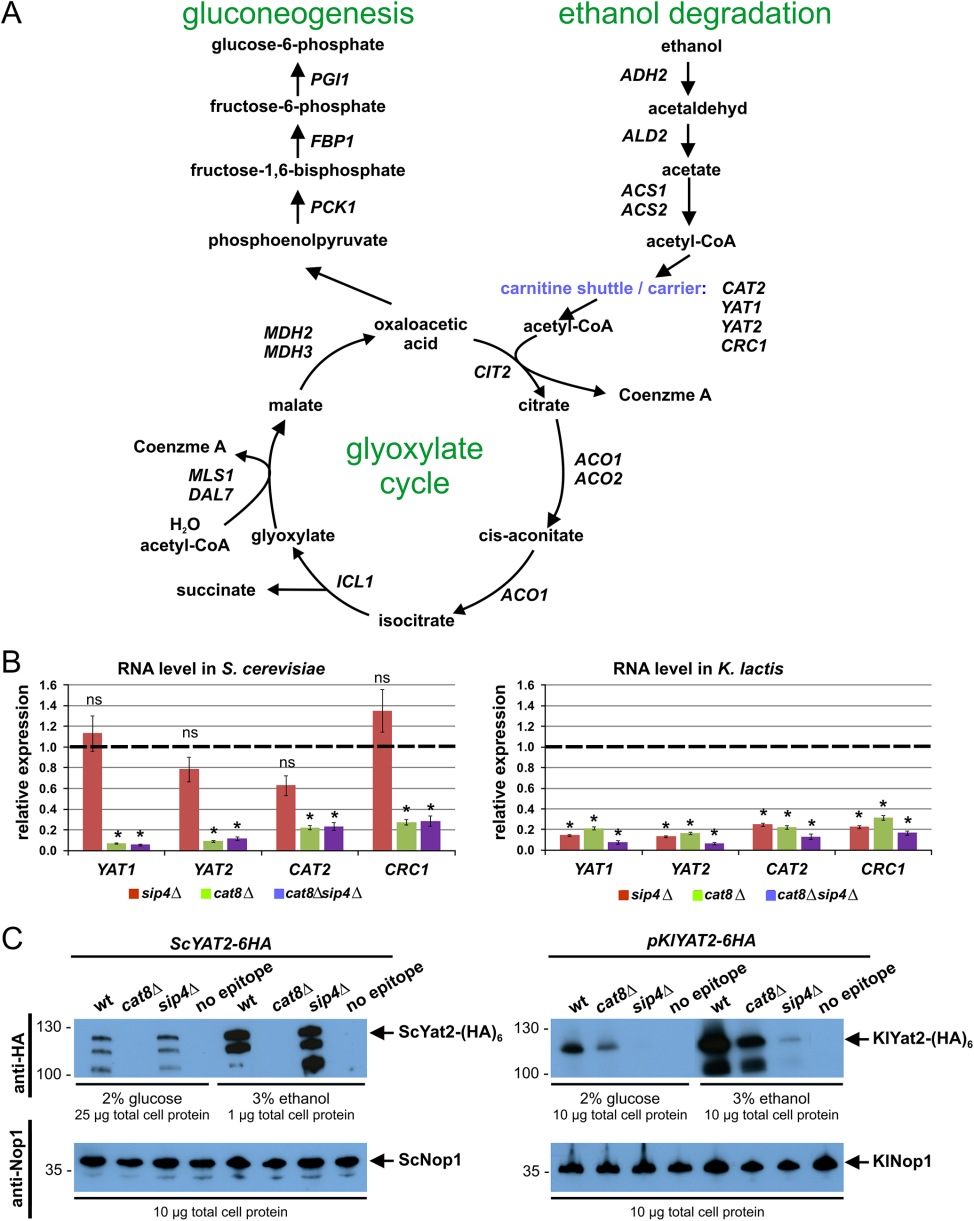
Sip4 is involved in expression of carnitine shuttle genes in *K*. *lactis*. (A) Schematic overview of metabolic pathways and key genes essential for the utilization of non-fermentable carbon source ethanol in yeast as found in the yeast genome database [33]. (B) RNA levels of genes related to the carnitine shuttle in *sip4*Δ, *cat8*Δ and *sip4*Δ*cat8*Δ mutants relative to congenic wild-type strains were determined by qRT-PCR in *S*. *cerevisiae* (left panel) and *K*. *lactis* (right panel). Cultures were shifted from 2% glucose to 3% ethanol medium for 2 hours at an OD_600_ of 0.8 to 1.0. Gene expression levels were normalized to the reference gene *HEM2* and quantified relative to wild-type levels (set to 1.0; dashed line) Data points and error bars represent mean values ± standard deviations obtained with three independent biological samples each measured in technical triplicates. Asterisks indicate statistically significant differences compared to wild-type (*t*-test; *P < 0.001; ns, not significant). (C) Protein levels of Yat2 in wild-type and mutant strains. In *S*. *cerevisiae* (left) the chromosomal *YAT2* gene was tagged with a (HA)_6_-epitope in wild-type and *cat8*Δ and *sip4*Δ mutant backgrounds (strains CMY196, CMY198 and CMY197), respectively. In *K*. *lactis* the strains JA6 (WT), yIG8 (*cat8*Δ) and JA6/DS4 (*sip4*Δ) were transformed with centromeric plasmid pCM68 (*KlYAT2-6HA*::pKATUC4) or empty vector (pKATUC4). Cultures were grown in glucose or ethanol medium and the indicated amounts of protein extracts were probed by Western blotting with anti-HA antibody (top panel). Nop1, detected with an anti-Nop1 antibody served as loading control (bottom panel). The position of KlYat2-(HA)_6_ (104.6 kDa) and ScYat2-(HA)_6_ (110.5 kDa) are indicated by arrows. Numbers refer to molecular markers in kDa.

To identify genes that require Sip4 to be expressed steady-state mRNA levels of a number of candidates relevant for growth on C2 carbon sources (Fig 3A) were compared in wild-type, *sip4*Δ, *cat8*Δ and *sip4*Δ*cat8*Δ mutants of *K*. *lactis* and *S*. *cerevisiae*. In both yeasts the selected genes are well established as being carbon source regulated having very low expression levels in glucose (data not shown). We also included the divergently transcribed genes *LAC4* (*KLLA0B14883g*) and *LAC12* (*KLLA0B14861g* **)** where a potential CSRE is located in the intergenic region (see below). The steady-state mRNA levels after a shift from glucose to ethanol for 2 hours were determined by quantitative real-time PCR (qRT-PCR) and are summarized in Table 1. Further statistic evaluation is provided as supplement (S2 and S3 Figs). The shift from the glucose medium to ethanol creates a situation of transient carbon depletion, because ethanol consumption requires the transcriptional induction of ethanol metabolic genes. Hence, wild-type cells enter a lag phase after the shift, and we compare non-proliferating cells of wild-type and mutants. Strikingly, the *K*. *lactis sip4* deletion markedly reduced transcript abundance of the glyoxylate cycle genes, *KlICL1* and *KlMLS1* encoding isocitrate lyase and malate synthase, respectively. For other genes, like those encoding acetyl-CoA synthases (*KlACS1*, *KlACS2*), citrate synthase (*KlCIT2*), aconitase (*KlACO1*), and a malate dehydrogenase (*KlMDH3*), transcripts were moderately reduced in the *Klsip4*Δ mutant. No significant reduction of *LAC4* and *LAC12* transcripts was detected. In the *Scsip4*Δ mutant none of the genes analyzed was substantially down-regulated. Rather several genes, like *PGI1*, *ADH2*, *MLS1* and the key gluconeogenic genes *FBP1* and *PCK1* were up-regulated, and up-regulation required ScCat8 and was not observed in the *Scsip4*Δ*cat8*Δ double mutant. Consistent with the mRNA data we found 3-fold and 2.5-fold higher protein levels of epitope-tagged ScPck1-(HA)_6_ and of ScMls1-(HA)_6_, respectively, in *Scsip4*Δ cells compared to wild-type (S4 Fig). Protein levels in the *Sccat8*Δ mutant were below detection. Apparently RNA levels correlate with protein levels under the conditions analyzed.

**Table 1:** Influence of *sip4*Δ and *cat8*Δ mutations on transcript abundances of selected orthologous genes.

		*S*. *cerevisiae*			*K*. *lactis*		
		RNA level [% of wild-type (SD)]					
*S*. *cerevisiae* gene	*K*. *lactis* ortholog	*sip4*Δ	*cat8*Δ	*sip4*Δ*cat8*Δ	*sip4*Δ	*cat8*Δ	*sip4*Δ*cat8*Δ
*PGI1*	*KLLA0E23519g*	**153** (9.5)	**181** (4.3)	**123** (1.4)	**n.m.**	**n.m.**	**n.m.**
*FBP1*	*KLLA0E01211g*	**229** (24.5)	**4.7** (1.1)	**3.8** (0.8)	**90** (9.2)	**107** (7.2)	**107** (2.1)
*PCK1*	*KLLA0A00484g*	**455** (23.2)	**6.3** (0.9)	**5.0** (0.8)	**79** (10.9)	**27** (2.4)	**50** (1.8)
*ADH2*	no ortholog	**187** (15.1)	**117** (3.9)	**92** (3.8)	**-**	**-**	**-**
*ALD2*	*KLLA0D10021g*	**87** (8.1)	**97** (7.2)	**99** (1.6)	**n.m.**	**n.m.**	**n.m.**
*ACS1*	*KLLA0A03333g*	**72** (3.4)	**12** (0.6)	**16** (0.3)	**40** (4.6)	**39** (3.3)	**27** (0.0)
*ACS2*	*KLLA0D17336g*	**120** (3.0)	**74** (1.2)	**58** (4.9)	**50** (4.1)	**11** (0.6)	**41** (3.4)
*CIT2*	*KLLA0F12760g*	**90** (4.9)	**53** (4.5)	**50** (0.1)	**53** (3.4)	**100** (6.7)	**102** (5.0)
*ACO1*	*KLLA0C17314g*	**101** (6.8)	**88** (10.2)	**99** (12.3)	**52** (3.6)	**36** (6.4)	**65** (2.5)
*ACO2*	*KLLA0C03432g*	**124** (8.2)	**100** (1.7)	**103** (6.3)	**83** (5.3)	**91** (7.6)	**132** (7.4)
*ICL1*	*KLLA0C08107g*	**100** (7.8)	**3.5** (0.4)	**3.7** (0.6)	**9.0** (1.1)	**2.9** (0.6)	**0.5** (0.1)
*MLS1*	*KLLA0F23914g*	**216** (4.4)	**6.9** (1.4)	**4.7** (0.9)	**12** (0.9)	**21** (3.8)	**3.8** (1.8)
*DAL7*	no ortholog	**92** (2.4)	**125** (5.9)	**86** (4.4)	**-**	**-**	**-**
*MDH2*	*KLLA0E07525g*	**102** (7.8)	**57** (4.6)	**48** (3.1)	**61** (6.8)	**259** (8.3)	**235** (4.6)
*MDH3*	*KLLA0F17050g*	**122** (4.4)	**178** (7.3)	**156** (15.7)	**52** (7.2)	**23** (1.7)	**12** (3.4)
*YAT1*	*KLLA0C04169g*	**109** (7.0)	**7,2** (1.1)	**5.6** (0.1)	**16** (3.1)	**26** (3.2)	**14** (3.4)
*YAT2*	*KLLA0D10637g*	**79** (6.6)	**9,0** (0.0)	**14** (2.1)	**14** (1.1)	**22** (3.4)	**12** (2.4)
*CAT2*	*KLLA0A02123g*	**103** (17.3)	**24** (0.4)	**25** (0.7)	**27** (5.8)	**28** (4.4)	**24** (0.7)
*CRC1*	*KLLA0C13431g*	**135** (8.6)	**32** (1.6)	**32** (2.7)	**24** (2.8)	**39** (6.0)	**23** (7.2)
*-*	*KLLA0B14883g*	**-**	**-**	**-**	**100** (5.0)	**43** (4.5)	**47** (5.3)
*-*	*KLLA0B14861g*	**-**	**-**	**-**	**72** (13.6)	**93** (5.1)	**103** (17.2)
*CAT8*	*KLLA0D01452g*	**96** (0.5)	**0.3** (0.0)	**0.3** (0.1)	**78** (9.7)	**0.0** (0.0)	**0.0** (0.0)
*SIP4*	*KLLA0F14322g*	**0.0** (0.0)	**4.0** (0.6)	**0.0** (0.0)	**0.1** (0.0)	**29** (6.8)	**0.0** (0.0)

The relative quantification of each transcript was calculated by the 2^ΔΔCt^ method normalized to *HEM2* as an internal control of gene expression. Values in percent relative to the wild-type are the average of three biological samples, each one quantified in triplicates. The standard deviation (SD) is shown in parenthesis. Statistical significance was measured using a one-way analysis of variance (ANOVA) and Tukey's post-hoc test to determine the difference between all pairwise comparisons. Data are summarized in S2 and S3 Figs).

In *K*. *lactis*, the *Klsip4Klcat8* double mutation had a stronger effect than either single mutation at genes like *KlICL1* and *KlMLS1* whereas at others (e.g. *KlACS2* and *KlACO2*) the strong influence of the *Klcat8* mutation was attenuated in the double mutation (Tukey's test; S3 Fig).

The results also reveal that in *K*. *lactis* but not in *S*. *cerevisiae* Sip4 is a key regulator of the carnitine shuttle genes (Fig 3A and 3B). Transcripts of orthologs of *YAT1* (*KLLA0C04169g* named *KlYAT1*), *YAT2* (*KLLA0D10637g* named *KlYAT2*), and *CAT2* (*KLLA0A02123g* named *KlCAT2*), that encode carnitine acetyltransferases and *CRC1* (*KLLA0C13431g* named *KlCRC1*), a mitochondrial acetyl-carnitine carrier gene, were reduced to the same extent of ≤20% in the *Klsip4*Δ and *Klcat8*Δ single mutants as well as in the double mutants (Fig 3B). In *S*. *cerevisiae*, a substantial reduction in mRNA levels of the corresponding genes was observed in the *cat8*Δ single and *cat8*Δ*sip4*Δ double mutants whereas the *Scsip4* deletion had no significant effect. Hence Cat8 is essential to activate those genes in both yeasts but KlCat8 may activate those genes indirectly via induction of *KlSIP4* (see below).

Again we addressed the question whether the differences in mRNA levels translate into differences in protein concentration by generating strains carrying epitope-tagged *YAT2-6HA* in wild-type, *sip4*Δ and *cat8*Δ backgrounds of both yeasts and the *YAT2* gene product was analyzed by Western blotting (Fig 3C; details in Material and Methods). We found a strong reduction of the ScYat2-(HA)_6_ protein in the *Sccat8*Δ mutant but wild-type levels in the *Scsip4*Δ mutant. In *K*. *lactis*, the Yat2-(HA)_6_ protein was undetectable in the *Klsip4*Δ mutant and reduced in the *Klcat8*Δ strain. Strikingly, in *S*. *cerevisiae* as well as in *K*. *lactis*, the difference between mutant and wild-type was not only obvious in ethanol but also in glucose-grown cells (note that in glucose the Yat2-(HA)_6_ protein concentration was much lower in all strains). Hence the transcription factors Cat8 and Sip4 also have an influence on gene expression in high glucose media.

In summary, we conclude that the inability of the *Klsip4*Δ mutant to assimilate C2 sources is at least partially due to the reduced transcription rates of glyoxylate and carnitine shuttle genes, which translate into reduced protein levels.

### KlSip4 binds to CSREs *in vivo*

To investigate whether KlSip4 or KlCat8 or both bind to genes differentially expressed in the mutants chromatin immunoprecipitation (ChIP) experiments were performed with strains expressing C-terminally (HA)_6_-epitope-tagged variants of KlSip4 and KlCat8 (Material and Methods) probing for promoters of the *KlICL1*, *KlMLS1* and *KlYAT2* genes, which contain putative CSREs. We also included the potential CSRE in the *LAC4* gene (CSRE_*LAC4*_) and one in the *KlSIP4* promoter.

**Fig 4:**
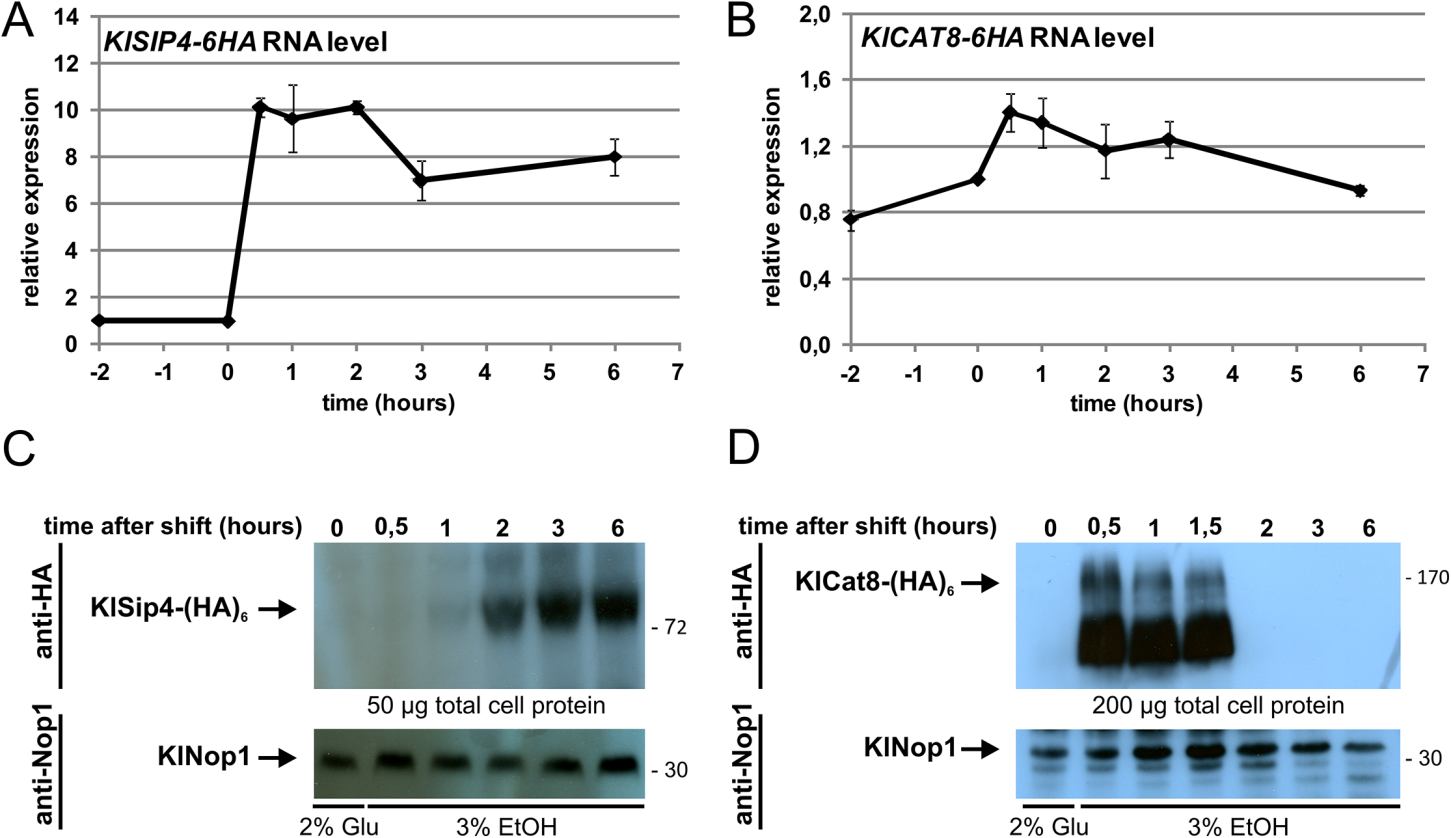
*KlSIP4-6HA* and *KlCAT8-6HA* RNA and protein levels after a shift to ethanol. (A and B) Time course of *KlSIP4-6HA* (A) and *KlCAT8-6HA* (B) gene expression. Wild-type cells expressing KlSip4-(HA)_6_ (JA6/S4HA) or KlCat8-(HA)_6_ (JA6/C8HA) were grown in glucose and shifted to ethanol medium at time zero. Samples were taken at the indicated time points, RNA was isolated and qRT-PCR was performed in triplicates. Fold changes relative to the time 0 sample were calculated by the 2^−∆∆CT^ method, normalized to the reference gene *KlHEM2*. (C and D) Time course of protein levels. In parallel to the RNA preparations (panel A and B) total protein extracts were prepared, separated by SDS-PAGE and analyzed by Western blotting using anti-HA antibody (top panel). Nop1, detected with an anti-Nop1 antibody served as loading control (bottom panel). The position of KlSip4-(HA)_6_ (89.1 kDa), KlCat8-(HA)_6_ (166.4 kDa) and KlNop1 (34.8 kDa) are indicated by arrows.

To obtain maximal occupancy of CSREs, the time after the medium shift at which the individual KlSip4-(HA)_6_ and KlCat8-(HA)_6_ proteins have the highest concentration was determined. Hence, the kinetics of accumulation of mRNA and protein was investigated for each of the transcription factors. Wild-type cells expressing KlSip4-(HA)_6_ or KlCat8-(HA)_6_ were shifted from glucose to ethanol medium and sampled at different time points for qRT-PCR (Fig 4A and 4B) and Western analyses (Fig 4C and 4D). While only little mRNA and no protein was detectable for KlSip4-(HA)_6_ as well as for KlCat8-(HA)_6_ during growth on glucose, a 10-fold-increase in *KlSIP4-6HA* mRNA level was observed thirty minutes after the shift to ethanol and the *KlSIP-6HA* mRNA level remained high for at least 6 hours (Fig 4A). Protein synthesis lagged behind and KlSip4-(HA)_6_ protein was detectable not earlier than one hour after medium shift (Fig 4C). Abundance increased until three hours after the shift and remained high thereafter. In contrast, the mRNA level for *KlCAT8-6HA* showed only a minor, transient increase after the shift to ethanol (Fig 4B), whereas the 166 kDa KlCat8-(HA)_6_ protein reached maximum abundance already after thirty minutes but became undetectable again two hours after the medium shift (Fig 4D). Hence, we shifted the *KlSIP4-6HA* strain for 3 hours and the *KlCAT8-6HA* strain for 30 min for the ChIP experiments. Note that even at maximum abundance, cells apparently contained much less Cat8-(HA)_6_ than Sip4-(HA)_6_ protein.

**Fig 5:**
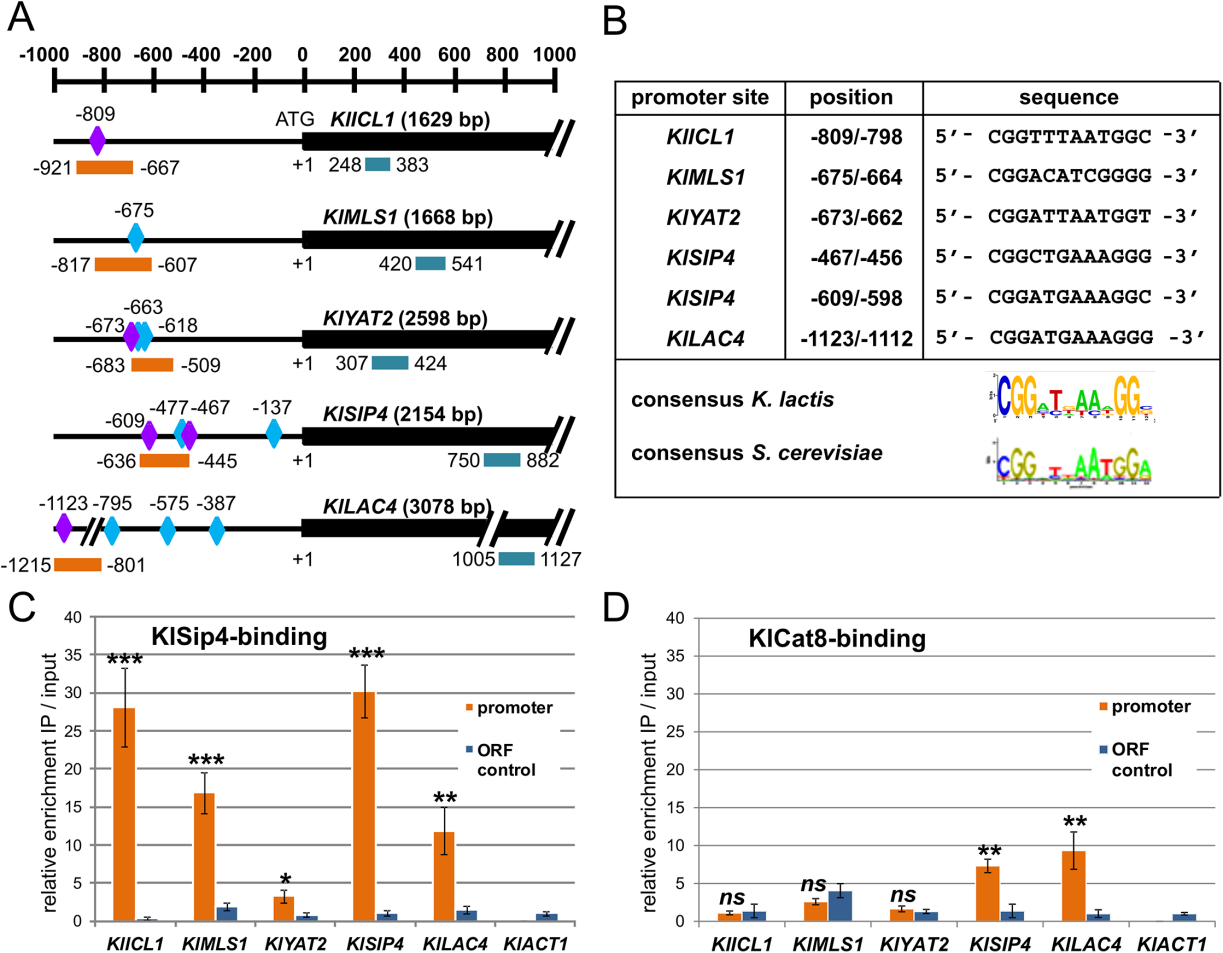
KlSip4-(HA)_6_ and KlCat8-(HA)_6_ binding to selected promoter regions. (A) Schematic overview of potential CSREs (diamonds) matching to the consensus sequences in promoter regions of putative target genes. The fragments amplified with promoter-specific (orange bars) and ORF-specific primer pairs (blue bars) for ChIP-qPCR analysis are indicated. Scale is 200 bp for one graduation. Positions are given relative to the ATG start codon (with +1). Blue diamonds indicate sequences conforming to the loose consensus sequence 5’- CGGNNNNNNGGN-3‘, purple diamonds to the more specific consensus sequence 5’-CGGNTKAAWGGN-3’. (B) CSREs in promoters of KlSip4 target genes and their distance from the ATG translation initiation site. A sequence logo for *K*. *lactis* was created using the Weblogo resource (http://weblogo.berkeley.edu/logo.cgi). [35]. The CSRE consensus sequence of *S*. *cerevisiae* [34] is shown for comparison. (C and D) ChIP-qPCR results indicating binding of KlSip4-(HA)_6_ (C) and KlCat8-(HA)_6_ (D) to selected promoters in strains JA6/S4HA and JA6/C8HA shifted to ethanol for 3 hours or 30 minutes, respectively. ORF-fragments amplified with specific primers pairs served as control for background binding and *KlACT1* for normalization to input-DNA. (Reference to mock ChIP with the untagged strain gave similar results as reference to inputs.) Data points and error bars represent mean values ± standard deviations obtained with three independent biological samples each measured in duplicates. The experiment was performed twice with similar results. Asterisks indicate statistically significant differences compared to input-DNA (*t*-test; *P<0.05; **P<0.01;***P < 0.001; ns, not significant).

Next, we searched in upstream regions of down-regulated genes (Table 1) for Sip4/Cat8 binding sites in *K*. *lactis*. The relatively soft consensus sequence 5’- CGGNNNNNNGGN-3‘ was used to locate potential CSREs within the respective promoters. Regions with such a motif (Fig 5A) were probed with appropriate primer pairs, and the corresponding ORFs lacking CSREs served as negative controls. All CSRE-containing promoter segments were enriched as compared to inputs and ORF sequences in chromatin immunoprecipitates of the tagged *KlSIP4-6HA* strain. Amplicons for the promoters of *KlICL1* and *KlMLS1* were most highly enriched (28- and 17-fold, respectively) (Fig 5C) whereas no enrichment was observed for the *KlACT1* promoter control lacking a CSRE consensus sequence. Apparently both, KlSip4-(HA)_6_ as well as KlCat8-(HA)_6_, bound effectively to the *KlSIP4* promoter (23-fold and 7.3-fold enrichment over input, respectively) and to the *LAC4-LAC12* intergenic region containing CSRE_*LAC4*_ whereas no significant enrichment of the other promoters tested was observed in the KlCat8-(HA)_6_ ChIP. Surprisingly, promoters of selected *S*. *cerevisiae* genes were also highly enriched in ScSip4-(HA)_6_ ChIP and only weakly in ScCat8-(HA)_6_ despite their dependence on ScCat8 but not on ScSip4 (S4 Fig, panel C).

An alignment of the six confirmed KlSip4-binding sites gave a CSRE consensus sequence 5’-CGGNTKAAWGGN-3’, which is very similar to the *S*. *cerevisiae* consensus [34](Fig 5B).

### The *KlSIP4* gene is induced by KlCat8 but not by KlSip4

**Fig 6:**
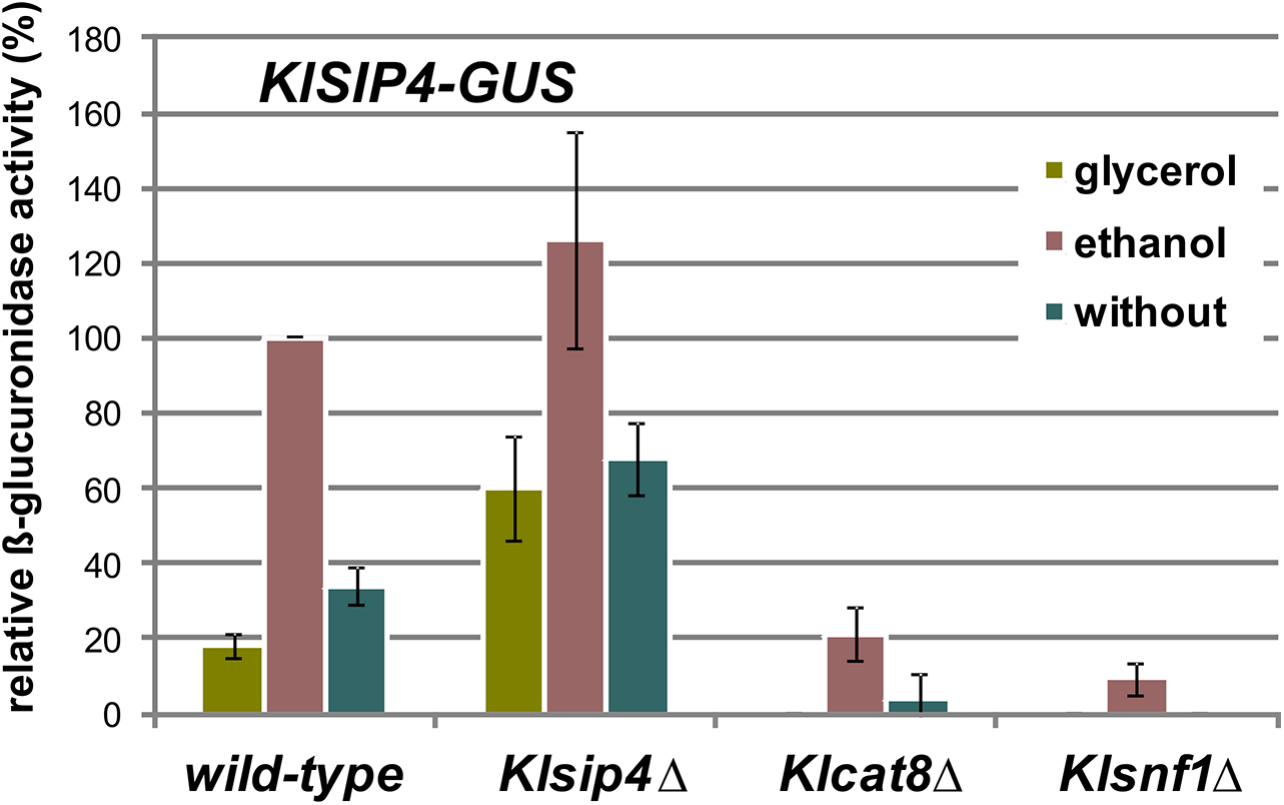
Influence of *Klsip4* and *Klcat8* mutations on *KlSIP4* promoter activity. The *KlSIP4* promoter was fused to the β-glucuronidase reporter gene on plasmid pLS2GUS, which was transformed into wild-type cells (JA6) and isogenic mutants *Klsip4*Δ (JA6/DS4), *Klcat8*Δ (yIG8) and *Klsnf1*Δ (JSD1R4), respectively. Transformants were pregrown in 2% glucose medium overnight and then shifted to medium with 3% glycerol, 2% ethanol or without any carbon source for 5 hours. β-glucuronidase activity (mU/mg protein) was assayed in whole cell extracts in three independent measurements and is given relative to the activity in ethanol-grown wild-type cells (100%) determined in parallel in each experiment. Mean values and standard deviation for the three measurements are presented.

Both, KlSip4 as well as KlCat8 apparently bind to the *KlSIP4* promoter, so we analyzed the influence of the *Klcat8*Δ and *Klsip4*Δ mutations on promoter activity. The 2-kb region upstream of the annotated translation start site was fused to the β-glucuronidase (GUS) reporter gene (plasmid pLS2GUS, see Material and Methods) and GUS expression of WT, *Klsip4*Δ and *Klcat8*Δ transformants was determined in different media (Fig 6). The *Klcat8*Δ mutation resulted in 80% reduction compared to the enzyme level of the wild-type strain grown in ethanol (100%), which is in line with the qRT-PCR data (Table 1). However, a *Klsip4*Δ deletion had no negative effect on *KlSIP4* promoter activity. On the contrary, the GUS levels were higher in the *Klsip4*Δ mutant than in wild-type in all media. Only background activity was measured for all strains in the presence of glucose (data not shown) and in a *Klsnf1* deletion strain. Hence, KlSip4 does not induce a positive feedback despite the fact that KlSip4 binds to its own promoter in wild-type cells, but rather seems to reduce to some extent the KlCat8-mediated activation of its promoter. Hence in this case KlCat8 appears to be the relevant activator while KlSip4 may function as a modulator by competing with KlCat8 binding (see Discussion). This situation contrasts with that at promoters where significant enrichment was only found in the KlSIP4-(HA)_6_ ChIP but not in the KlCat8-(HA)_6_ ChIP [36]. Hence we conclude that by binding to CSREs, KlSip4 can function as an activator in cases where KlCat8 binding is not detected or as a modulator of the KlCat8 activator in cases were both can bind.

### The role of CSRE_*LAC4*_ in regulating the *LAC* genes

**Fig 7:**
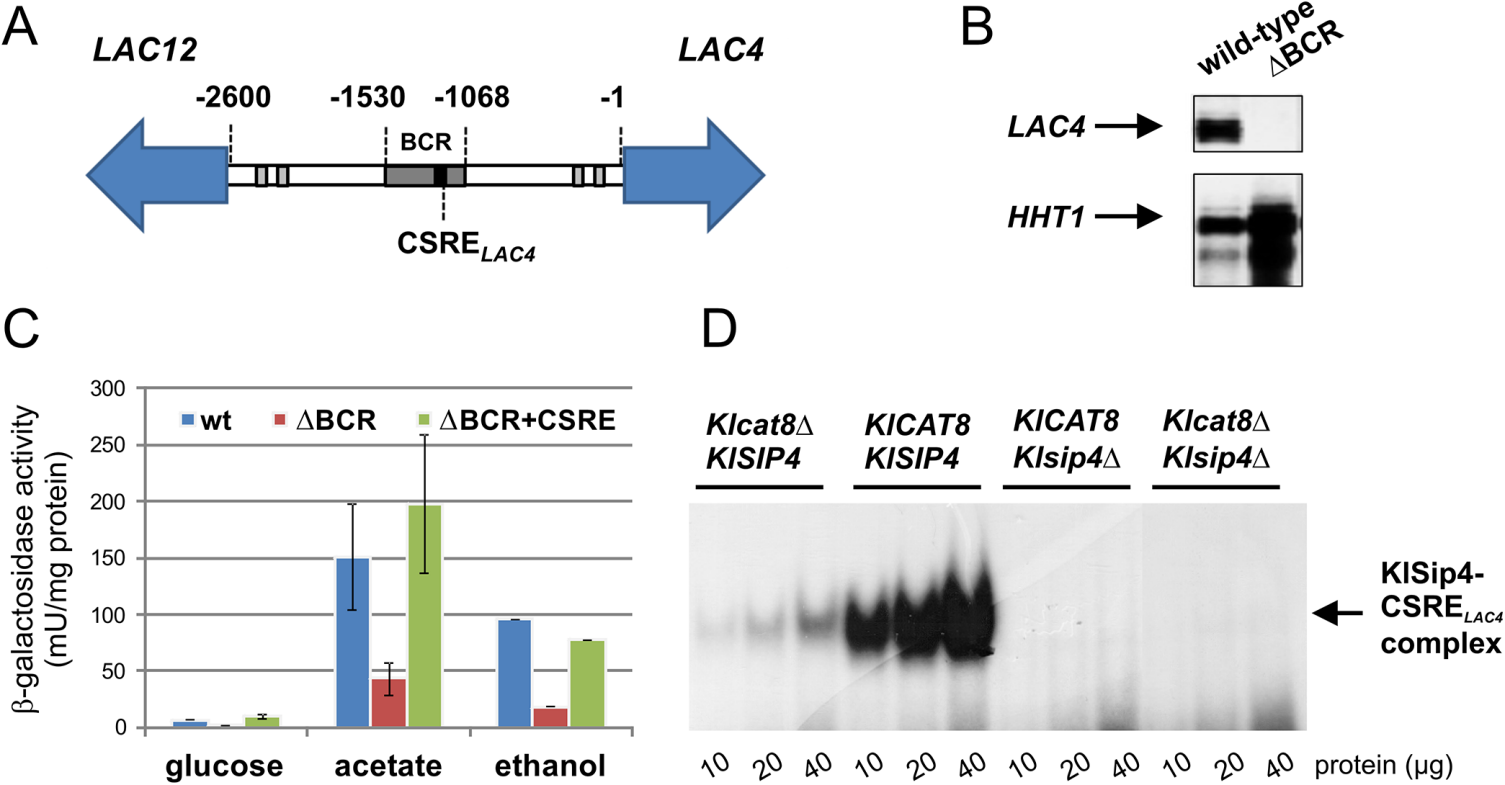
The basal control region (BCR) and CSRE_*LAC4*_ regulate transcription of the *LAC4* gene. (A) Intergenic region between the divergently transcribed genes *LAC12* and *LAC4* containing the basal control region (BCR, shaded in dark gray) and CSRE_*LAC4*_ (black box). Four binding sites for the transcription activator KlGal4 are indicated by light grey boxes. Distances are given relative to the *LAC4* ATG. (B) Non-induced *LAC4* mRNA levels in wild-type and a BCR deletion mutant (ΔBCR; strain JA6/LR2). Total RNA was isolated from cells grown in 3% glycerol and analyzed by quantitative S1 nuclease mapping as described previously [39]. Histone H3 RNA (*HHT1*, lower lanes) served as loading control.(C) Transcription activation function of CSRE_*LAC4*._ Non-induced *LAC4* expression was determined by β-galactosidase measurements in extracts from wild-type (JA6), JA6/LR2 (ΔBCR) and JA6/LR2K (ΔBCR::CSRE_*LAC4*_) strains grown in synthetic complete medium containing 2% glucose, 2% acetate or 2% ethanol. (D) Dependence of CSRE_*LAC4*_–protein complex formation on *KlCAT8* and *KlSIP4*. 10, 20 or 40 μg of S100 protein extracts from wild-type (JA6), *sip4*Δ (JA6/DS4), *cat8*Δ (yIG8) or *sip4*Δ*cat8*Δ (yIG8/DS4) cells grown in synthetic complete medium with 3% glycerol were used for EMSA with a labeled CSRE_*LAC4*_ oligonucleotide (~80,000 cpm).

CSRE_*LAC4*_ is another site, which binds both KlCat8 and KlSip4. It is located about midway between *LAC12* and *LAC4* encoding the key proteins in lactose utilization, lactose permease and β-galactosidase, respectively (Fig 7A). These genes are separated by an unusually large 2.6 kb intergenic region and are induced in response to lactose by the transcription activator KlGal4 (or Lac9, the *K*. *lactis* homolog of Gal4) [37–39]. However, basal expression of the *LAC* genes under non-inducing, non-repressing growth conditions is KlGal4-independent [40]. Deletion of a segment (-1530 to -1068 upstream of the *LAC4* ATG), which includes the CSRE consensus sequence, reduced the *LAC4* transcript in the absence of inducing sugars to undetectable levels so this region was named Basal Control Region (BCR) (Fig 7A and 7B). Mutation of CSRE_*LAC4*_ in the wild-type context reduced basal β-galactosidase activity to only 50% [41] indicating that loss of CSRE_*LAC4*_ mediates only partly the effect of the BCR deletion.

To address the question whether the CSRE_*LAC4*_ alone was able to enhance basal expression in a strain lacking the BCR (JA6/LR2), a 20-bp oligonucleotide encompassing CSRE_*LAC4*_ was integrated at the site of the deletion and *LAC4*-encoded β-galactosidase activity was determined. The CSRE_*LAC4*_ was capable of restoring basal activity to wild-type levels (Fig 7C) confirming that CSRE_*LAC4*_ could function as an upstream activating sequence regulating expression of *LAC4* under non-fermentative growth conditions. Notably, as mentioned above, in the wild-type promoter context the deletion of this motif had only a moderate effect on *LAC4* expression. Apparently, other elements within the BCR are capable of compensating loss of CSRE_*LAC4*_.

Electrophoretic mobility shift assays (EMSA) were performed with *K*. *lactis* soluble protein extracts (see Material and Methods) and a double-stranded CSRE_*LAC4*_ oligonucleotide to detect a CSRE_*LAC4*_-protein complex. In the wild-type background (*KlCAT8 KlSIP4*) a prominent complex was detected, which was strongly reduced in the *Klcat8*Δ strain and absent in both strains lacking *KlSIP4* (Fig 7D). The intensity of the complex was regulated by the carbon source (S5 Fig) correlating with the activity of the *KlSIP4* promoter (Fig 6). These results indicate that the band corresponds to a KlSip4-CSRE_*LAC4*_ complex. The presence of KlSip4 in the complex was confirmed in the strain expressing the epitope-tagged KlSip4-(HA)_6,_ where the anti-HA antibody produced a super-shift in the EMSA (data not shown). It can be excluded that the complex also contains KlCat8 since its mobility is not affected by the absence of KlCat8.

## Discussion

### The CSRE-binding factors Sip4 and Cat8

Transcription factors with similar or identical DNA-binding specificities are common in gene regulatory networks and their mutual influence depends on their specificities and their relative concentrations. Here we have analyzed the role of Cat8 and Sip4, both of which bind to carbon source-responsive elements and are members of the Gal4 family of Zn(II)_2_Cys_6_ transcription factors. This protein family is conserved in the fungal kingdom of life and has been characterized in several fungal species, most detailed in *S*. *cerevisiae* [18,20]. Remarkably, *SIP4* homologs are found only in *Hemiascomycota*.

Our comparative study in *S*. *cerevisiae* and *K*. *lactis* revealed a novel role for the transcription factor Sip4. Differential expression in *sip4*Δ mutants and wild-type revealed that *K*. *lactis* Sip4 is essential for regulating the glyoxylate cycle and the carnitine shuttle, a function that is not observed in *S*. *cerevisiae*. This explains why, unlike *S*. *cerevisiae*, a *K*. *lactis sip4* deletion mutant is unable to grow on C2 carbon sources like ethanol or acetate.

**Fig 8:**
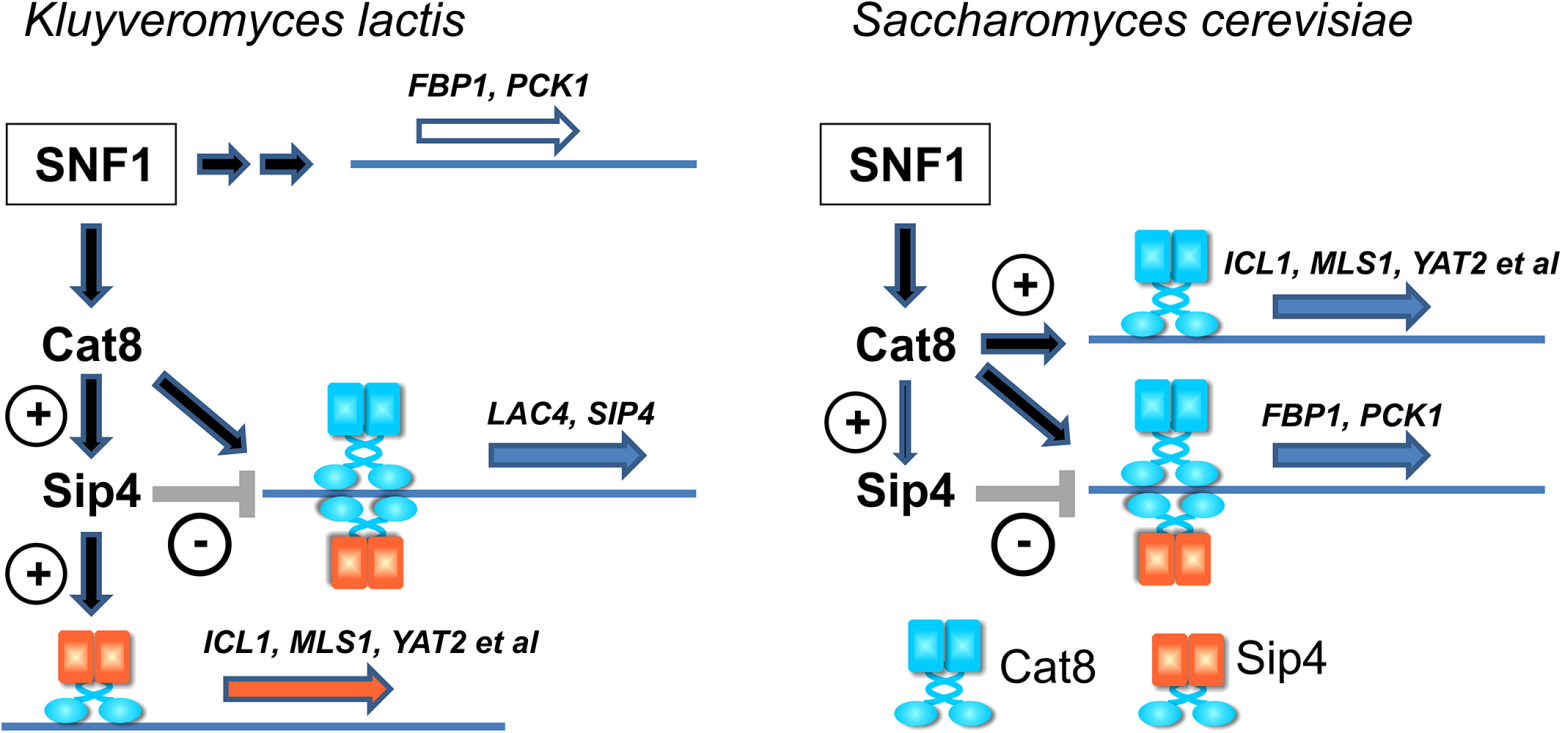
Comparison of the SNF1-Cat8-Sip4 regulatory network in *K*. *lactis* and *S*. *cerevisiae*. Schematic representation of regulation by the CSRE-binding transcription factors Cat8 and Sip4 for selected orthologous genes. The cartoons symbolize the similarity in the DNA-binding domains (blue ovals), and the differences in the rest of the proteins (blue/yellow rectangles).

Also in contrast to *S*. *cerevisiae*, expression of gluconeogenic genes in *K*. *lactis* requires neither KlCat8 nor KlSip4 and hence the single and double mutants are not impaired in utilizing the non-fermentable C3 carbon source glycerol. The *Sccat8*Δ mutant, which originally has been isolated in a screen for factors involved in glucose derepression [16], does not support growth on glycerol because expression of key gluconeogenic enzymes depends on ScCat8. Hence, a rewiring of the Snf1-Cat8-Sip4 regulatory network occurred during the divergent evolution of *K*. *lactis* and *S*. *cerevisiae* (Fig 8).

The absence of a phenotype in a *Scsip4* deletion mutant has suggested genetic redundancy. Indeed, a new zinc cluster protein, Ert1 encoded by the *YBR239C* gene, was recently shown to genetically interact with *ScSIP4* [42]. In *K*. *lactis*, *a* putative *ERT1* homolog exists (*KLLA0F10417g*), but the phenotype of the *Klsip4*Δ single mutant shows that neither this nor the *KlCAT8* gene is able to replace KlSip4 function.

### KlSip4 acts downstream of KlCat8

In *K*. *lactis* overexpression of *KlSIP4* can suppress the growth defect on ethanol of the *Klcat8*Δ mutant indicating that KlSip4 has a role downstream of KlCat8. Such a regulatory cascade SNF1-Cat8-Sip4 has also been proposed for *S*. *cerevisiae* [28]. However, in contrast to *K*. *lactis*, there is no information about an essential role of ScSip4 as transcription activator in a *ScCAT8* positive genetic background.

Here we have shown that the concentration of *KlSIP4* mRNA is reduced 3- to 5-fold in a *Klcat8* mutant. Conversely, *KlCAT8* gene expression is not affected by the *Klsip4* deletion (Table 1) and multicopy *KlCAT8* cannot suppress the impaired growth on C2 carbon sources of the *Klsip4*Δ mutant [43]. Hence, Cat8 controls Sip4 but not *vice versa*. The *KlSIP4* promoter contains CSREs and KlCat8 binds to the promoter *in vivo*, as shown by ChIP, indicating that the *KlSIP4* gene is a direct target of the transcription activator KlCat8. We conclude that activation of the *KlSIP4* gene is an important function of KlCat8 in *K*. *lactis*.

### KlSip4, a positive and negative regulator of gene expression

The inability of the *Klsip4*Δ mutant to assimilate C2 carbon sources like ethanol (or acetate) can be explained by lack of up-regulation of several genes encoding essential enzymes in the pathway. *KlACS1* and *KlACS2* encoding acetyl-CoA synthases, the key enzyme converting ethanol or acetate to acetyl-CoA, were only moderately reduced in the *Klsip4* mutant whereas expression of all four genes of the carnitine shuttle, *KlYAT1*, *KlYAT2*, *KlCAT2* and *KlCRC1* and the key genes of the glyoxylate cycle *KlICL1* and *KlMLS1* was severely reduced. The carnitine shuttle is essential to mobilize acetyl-groups over intracellular membranes, both in yeast and in animal cells, since membranes are impermeable to acetyl-CoA. The acetyl moiety is transferred to carnitine to form acetyl-carnitine, which can then be transported via the carnitine carrier. Only in *K*. *lactis* Sip4 is required to activate the genes related to the carnitine shuttle. Differences in mitochondrial activity resulting in differences in the compartimentation of acetyl-CoA between *K*. *lactis* and *S*. *cerevisiae* may relate to the fact that the function of Sip4 to provide acetyl-CoA is not conserved in *S*. *cerevisiae*. In *S*. *cerevisiae* grown in glucose most of the glycolytic pyruvate is converted into acetyl-CoA in the cytosol whereas in *K*. *lactis*, pyruvate is primarily metabolized by the mitochondrial pyruvate dehydrogenase complex giving mitochondrial acetyl-CoA [44]. Hence, not only under carbon limitation but also in glucose medium the acetyl-moiety has to cross the mitochondrial membrane to fuel the nuclear/cytoplasmic pool. Consistently, we find that at least the carnitine acetyltransferase Yat2 is up-regulated by KlCat8 and KlSip4 also in glucose medium.

The enzymes of the glyoxylate cycle isocitrate lyase and malate synthase, encoded by *ICL1* and *MLS1*, respectively, catalyze anaplerotic reactions, which become important upon carbon limitation. Both enzymes, which in yeast are located in the cytosol, provide metabolites that condense with cytosolic acetyl-CoA forming malate or citrate, respectively, that can then be transported to mitochondria to enter the TCA cycle or replenish the oxaloacetate pool for biosynthetic purposes (Fig 3A). Malate synthase, together with non-mitochondrial citrate synthase, is crucial in regulating the distribution of acetyl-CoA between mitochondria and the glyoxylate cycle [45]. The glyoxylate cycle not only fuels biosynthesis but also plays an important role in NADH production [45]. Hence, KlSip4 holds a key position between anabolic and catabolic reactions in *K*. *lactis* by regulating all these genes.

A major carbon sink in yeast is biosynthesis of cell wall and carbon storage carbohydrate synthesis. Gluconeogenesis provides the required C6 metabolites in the absence of a fermentable carbon source. Remarkably, in *S*. *cerevisiae* the genes *FBP1*, *PCK1* and *MLS1* encoding key gluconeogenic enzymes were up-regulated in the *Scsip4*Δ mutant. We hypothesize that at these genes ScSip4 competes with ScCat8 for CSRE binding and hence functions as a negative regulator of promoter activity (Fig 8). This would be a way to modulate gluconeogenesis via the ratio between Cat8 and Sip4, which to our knowledge is unexplored so far.

KlSip4 binding to several putative CSREs was confirmed by ChIP including two CSREs in its own gene and a CSRE in the *LAC4* promoter. A deletion of *KlSIP4* had a moderate positive impact on promoter activity in these cases indicating that KlSip4 like ScSip4, can function as a negative regulator of transcription. Competition of KlCat8 and KlSip4 for the same CSRE is again likely to be responsible for this effect since both KlCat8 and KlSip4 were shown to associate with the CSREs at these sites. For *KlSIP4* gene regulation, the negative feedback may be important for preventing overshooting of the regulatory cascade. For the *LAC4* gene, responsiveness to lactose induction may depend on the balance between KlSip4 and KlCat8. Lactose has to be hydrolyzed to produce the effective inducer, intracellular galactose. To initiate induction the basal expression of *LAC4* encoding the lactase enzyme is crucial and determines the lag phase until the induction process is self-amplified via the galactose regulon [15].

In *S*. *cerevisiae* as well as in *K*. *lactis*, Sip4 accumulates under starvation conditions when Cat8 levels are reduced. Hence the ratio between the two is shifted in favor of Sip4 upon prolonged starvation. For those promoters where Sip4 negatively affects Cat8-dependent transcription activation Sip4 would be in an optimal position to signal growth-inhibitory starvation conditions where gene induction would be needless or ineffective. What prevents KlSip4 from activating transcription from such binding sites remains to be unraveled.

We propose that the balance between Cat8 and Sip4 is an important element in assuring cellular homeostasis. The fermentative life style of *S*. *cerevisiae* is associated with glucose repression of many genes and the response to starvation requires massive reprogramming of gene activity. Part of this is regulated by the transcription activator Cat8, which is transcriptionally and post-translationally regulated by SNF1 [22,23] and controls a large set of genes. The genetic network controlled by Snf1 plays a key role in the diauxic shift, which has recently been compared in 15 yeast species including *K*. *lactis* and *S*. *cerevisiae* [46]. The results revealed that the whole genome duplication that has shaped the *S*. *cerevisiae* genome has accelerated the evolution of gene regulation resulting in extensive re-wiring of the genetic network controlling adaptation to glucose deprivation.

In *K*. *lactis*, a higher mitochondrial activity under non-starvation conditions implies a less dramatic metabolic shift when carbon becomes scarce but leads to a higher requirement of building blocks for gluconeogenic growth because of a low glycolytic flux. The genes activated by *KlSIP4* encode enzymes that feed into the TCA cycle. We hypothesize that Sip4 has an important role in providing substrates for gluconeogenesis and cell growth when TCA cycle intermediates become scarce.

## Material and Methods

### Yeast media, growth conditions and transformation methods

*S*. *cerevisiae* and *K*. *lactis* were grown in rich (YPD) or synthetic complete (SC) media [47]. Cells pregrown over-night in liquid YP medium (phenotype analysis) or SD medium (shift experiments) supplemented with 2% glucose were diluted into fresh medium to OD_600_ 0.3 and grown at 30°C approximately to OD_600_ 0.8 – 1.0. For shift experiments, cells were washed twice in sterile water, transferred to SC media containing 3% ethanol as sole carbon source and grown for the indicated times at 30°C. Phenotypic characterization was performed on SC medium supplemented with the required amino acids and/or bases and containing 2% glucose (w/v) or 2% ethanol, 2% glycerol or 2% acetate as carbon source in serial 10-fold dilutions. Plates were incubated at 30°C for 4 days. Transformation of *S*. *cerevisiae* with plasmid DNA or polymerase chain reaction (PCR) products was performed by the lithium acetate procedure [48]. *K*. *lactis* cells were transformed by the polyethylen glycol method [49] modified by Dohmen et al. [50]. Selection for reversion from prototrophy to uracil auxotrophy was performed on SC plates containing uracil, 2% glucose and 0.5 μM 5-fluoroortic acid (FOA) as described by [51].

### Sequence analyses, alignment, and phylogenetic tree

Orthologues for *Kluyveromyces lactis* Sip4 (NCBI accession number CAE00852.1) and Cat8 (XP_453133.1) were found using the database: OrthoDB (http://cegg.unige.ch/orthodb5) [52]. Multiple sequence alignments of full-length amino acid sequences of: *Saccharomyces cerevisiae* Sip4 (CAA89382.1); *Ashbya gossypii* AFR096Wp (NP_985643.2); *Candida glabrata* CAGL0L03377g (CAG61889.1); *Zygosaccharomyces rouxii* ZYRO0G15136p (CAR29706.1); *Lachancea thermotolerans* KLTH0D03564p (XP_002552880.1); *Vanderwaltozyma polyspora* Kpol_1016p20 (XP_001643936.1); *Saccharomyces cerevisiae* Cat8 *(CAA55139*.*1)*; *Ashbya gossypii* ABL121Cp (NP_982826.2); *Candida glabrata* CAGL0M03025g (XP_449478.1); *Zygosaccharomyces rouxii* ZYRO0G14278g (XP_002498603.1); *Lachancea thermotolerans* KLTH0C03762p (XP_002552389.1); *Aspergillus nidulans* FacB (CBF88979.1); *Aspergillus niger* FacB (XP_001392773.1) and *Aspergillus oryzae* FacB (XP_001727529.2) were constructed using ClustalW and displayed with BioEdit (Version 7.2.5) [53]) using the BLOSUM62 matrix and a 50% threshold for shading. Phylogenetic analysis was performed using MegAlign Pro in Lasergene (SeqMan NGen®. Version 12.0. DNASTAR. Madison, WI.) with the default parameter setting, using the full-length amino acid sequence. The evolutionary relationship predicted from the multiple sequence alignment is presented as a phylogram with branch lengths proportional to the distance between sequence pairs. The scale below the tree indicates the number of amino acid substitutions per 100 residues for protein sequences.

### Strain and plasmid constructions

Yeast strains and primers are listed in S1 and S2 Tables. In *S*. *cerevisiae*, defined *sip4*Δ and *cat8*Δ null alleles were obtained after transformation of PCR fragments generated with template plasmids YDp-KlL or YDp-KlU [54] containing the marker genes *KlLEU2* or *KlURA3*, respectively, and appropriate knockout primer pairs (S2 Table). For PCR-mediated C-terminal tagging with a 6HA epitope, plasmid pYM3 and S3/S2-derived primers were used as described [55,56]. The plasmid pGP3 containing the *K*. *lactis KlSIP4* gene was selected from a KEp6 based genomic library [57] as decribed in the results section. *sip4*Δ deletion strains JA6/DS4 and yIG8/DS4 were obtained by two-step gene disruption using plasmid pDS4, which carries the *KlSIP4* fragment (-325 / +2994 deleted between -18 and +2182) in a pBR322 backbone with the *ScURA3* gene (pBRURA). *Xho*I digested pDS4 was inserted at the *KlSIP4* locus in the first step. In the second step, the intact *KlSIP4* gene and the inserted bacterial vector sequences including the *ScURA3* marker were eliminated via recombination between repeats selecting for uracil auxotrophy on 5-FOA plates. The resulting strains carried the indicated deletion at the chromosomal *Klsip4* locus, which was confirmed by Southern analysis. To generate 6HA-epitope tagged versions of the *KlSIP4 and KlCAT8* genes (*KlSIP4-6HA*, *KlCAT8-6HA*) fragments containing the 6HA-epitope fused to the respective C-termini and a downstream *KlTRP1* gene were generated by PCR using primers S3-KlSIP4/S2-KlSIP4 or S3-KlCAT8/S2-KlCAT8, respectively, and pYM3 [55] as template. The PCR fragments were integrated via homologous recombination in *S*. *cerevisiae* into plasmids pGP3 or pGID1 [21], giving pGP3HA (*KlSIP4-6HA*::KEp6) and pCM66 (*KlCAT8-6HA*::KEp6), respectively. Epitope tagging of the chromosomal genes in strains JA6/S4HA and JA6/C8HA was carried out by gene replacement in strain yIG8 (*Klcat8*Δ) or JA6/DS4 (*Klsip4*Δ) upon transformation of *Xag*I/*Bpu*1102I digested pGP3HA or *Hin*dIII digested pCM66, respectively. The HA-tagged versions of the *KlSIP4* and *KlCAT8* genes (*KlSIP4-6HA* and *KlCAT8-6HA*) were shown to be fully functional by complementing the growth defect of the respective mutants (S6 Fig). In addition, the plasmid pGP3HA was integrated at the *LAC4* locus of wild-type, *Klsip4*Δ, *Klcat8*Δ and *Klsnf1*Δ cells giving strains JA6/SIP4HA, DS4/SIP4HA, yIG8/SIP4HA and JSD1R4/SIP4HA carrying a second *KlSIP4* gene copy. pCM68 containing *KlYAT2-6HA* in the centromeric pKATUC4 vector [58] was created from three PCR products using the In-Fusion cloning strategy [59]: (i) The *6HA-KlTRP1* cassette was amplified from pYM3 [55] using primers TRP-IF2_FW and TRP-IF2_RV; (ii) the *KlYAT2* coding region (without STOP codon) including a 1000 bp upstream segment flanked by a *Sph*I site and a 1000 bp downstream segment of *KlYAT2* flanked by a *Sac*I site was amplified from genomic DNA of *K*. *lactis* strain JA6, using primer pairs KlYAT2-IF1_FW / KlYAT2-IF1_RV and KlYAT2-IF3_FW / KlYAT2-IF3_RV, respectively. The three PCR fragments were gel purified, mixed in equimolar concentrations, added to the *Sph*I/*Sac*I-cleaved pKATUC4 vector and incubated at 50°C for 15 minutes in 10 μL 1X In-Fusion™ enzyme mix (Clontech Laboratories, Inc.). 2.5 μl of the In-Fusion™ reaction was transformed into 50 μl of competent Stellar™ *E*. *coli* cells (Clontech Laboratories, Inc.). Ampicillin-resistant clones were characterized by *Sph*I/*Sac*I digestion (Thermo Fisher Scientific). The strain JA6/LR2 carrying the LR2 deletion (Δ-1530 to -1068) was generated in a series of *LAC4* promoter deletions using *Xba*I-cleaved plasmid pLR2 as described [39,60]. To construct strain JA6/LR2K a double-stranded oligonucleotide (5'-AATTCTCGGATGAAAGGGGGAATT-3') with CSRE_*LAC4*_ was inserted at the site of the deletion in pLR2 to give pLR2K. pLR2K was digested with *Xba*I and transformed into strain JA6/DL4 (*lac4*::*ScURA3*). Lac^+^ Ura^-^ transformants were selected and shown to restore an intact *LAC4* gene, the LR2 deletion containing CSRE_*LAC4*_ replacing the wild-type *LAC4* promoter. Correct integration was confirmed by DNA sequencing and Southern analysis. The *KlSIP4* promoter activity was analyzed by fusing a PCR fragment (generated with primers SIP4-P-for and SIP4-P-rev) with the *E*. *coli* glucuronidase (GUS) reporter gene and inserting the fusion into *K*. *lactis* centromeric vector pKATUC4 [58] giving pLS2GUS.

### RNA preparation and quantitative RT-PCR analysis (qRT-PCR)

Wild-type and deletion strains were grown in SC media with 2% glucose to OD_600_ 0.8–1.0, washed twice in water, shifted to SC media containing 3% ethanol and grown for 2 hours. Total RNA was isolated using the EURx GeneMATRIX Universal RNA Purification Kit (Roboklon). The purity and concentration was determined by spectrophotometry (NanoPhotometer, Implen). cDNA was reverse-transcribed from 500 ng total RNA using the RevertAid First Strand cDNA Synthesis Kit (ThermoScientific). The qRT-PCR reactions were performed on an iCycler iQ5 Real-Time PCR Detection System (Bio-Rad) using 2x iQ SYBR GreenSupermix (Bio-Rad) and gene-specific primers (S2 Table). The following cycling parameters were used: 95°C for 10 min followed by 50 cycles of 95°C for 15 s, 55°C for 15 s and 72°C for 30 s. To confirm the specificity of each PCR an additional melting curve analyses were performed by heating reactions from 55°C to 95ºC in 81 steps of 0.5°C with 10 s intervals. Threshold cycles (Ct) were calculated automatically using the Bio-Rad iCycler iQ5 software V2.1. To ensure the absence of random and genomic DNA contaminations no template controls and a reaction with no reverse transcriptase were included on each plate. Reference genes *HEM2*, *RPS26A* and *RDN18* for *S*. *cerevisiae* and *HEM2*, *ALG9* and *IPP1* for *K*. *lactis*, selected from a set of reference genes described in *S*. *cerevisiae* as non-regulated housekeeping genes [61] were used as internal controls to normalize transcript abundance. The open access software application LinRegPCR V12.18 [62,63] was used to calculate the specific amplification efficiencies from all single fluorescence curves in one amplicon group via linear regression in the exponential part of the fluorescence curves. The relative quantification method including efficiency (E) correction published by Pfaffl (2004) was used to calculate the ratio of a target mRNA between wild-type and mutant: $ratio=\frac{(E\;target)\Delta Ct\;wildtyp-mutant}{(Ereference)\Delta Ct\;wildtype-mutant}$ratio = (E_target gene_) ΔCt_wildtype - mutant_ / [(E_reference gene_) ΔCt_wildtype - mutant_]. Presented results are mean values from three independent yeast cultures each analyzed in three technical replicates.

### Western blot analysis

Total protein extracts were prepared from 50 ml cultures grown in SC medium supplemented with the appropriate carbon source. Cell pellets were resuspended in 400 μl B60 buffer (50 mM HEPES pH 7.3, 60 mM sodium acetate, 5 mM magnesium acetate, 0.1% Triton X-100, 10% glycerol, 1 mM sodium fluoride, 20 mM glycerophosphate, 1 mM DTT, 1X Complete Mini Protease Inhibitor Cocktail (Roche)). An equal volume of glass beads was added and cells were disrupted using a vortexer for 4 x 4 minutes, followed by centrifugation at 14,000 rpm and 4°C for 5 minutes. The supernatant was transferred to a new tube and centrifuged at 14,000 rpm and 4°C for 20 minutes. Protein concentrations were determined by the method of Bradford [64]. Supernatants from each extraction were analyzed by SDS/PAGE and Western blot using anti-HA (F-7) (Santa Cruz) antibodies and calibrated with anti-Nop1 (yA-17) (Santa Cruz) antibodies recognizing yeast Nop1 in *S*. *cerevisiae* and *K*. *lactis* as loading control. Blots were probed with secondary antibodies conjugated to horseradish peroxidase (Jackson Immuno Research) and detected by chemiluminescence and exposed to X-ray films.

### Chromatin immunoprecipitation (ChIP) and quantitative PCR (qPCR)

*S*. *cerevisiae* and *K*. *lactis* strains with 6HA epitope-tagged *SIP4* and *CAT8* genes were grown in SC medium with 2% glucose to an approximate OD_600_ of 0.8, washed twice in water, transferred to SC media containing 3% ethanol as a sole carbon source and grown for 3 or 0.5 hours, respectively. ChIP was performed as described in Lefrancois *et al* [65] with one exception. DNA shearing was performed using a M220 Focused-ultrasonicator (Covaris). Immunoprecipitated DNA was analyzed by qPCR on a CFX real-time PCR detection system (Bio-Rad) and the appropriate Bio-Rad CFX Manager software V.2.0. Primer for gene-specific qPCRs were designed to cover promoter regions and are listed in S2 Table. The amount of immunoprecipitated DNA was calculated relative to the input DNA using the 2^-ΔΔCt^ method [66]. Experiments were performed in biological triplicates and analyzed in two technical replicates.

### Reporter gene assay / enzyme measurements

To measure β-galactosidase or β-glucuronidase activity cultures were grown in YPD overnight, shifted to SC media with 3% glycerol, 2% ethanol, 3% acetate or without any added carbon source at OD_600_ 0.1 to induce transcription and grown further to OD_600_ = 0.8 to 1.6. Cells were harvested and glass bead-disrupted in β-galactosidase buffer (5.0 mM Tris-HCl pH7.8; 5% glycerol; 10 mM KCl) or β-glucuronidase buffer (10 mM β-mercaptoethanol; 10 mM Na_2_EDTA; 0.1% sodium lauroyl sarcosinate; 0.1% Triton X-100; 10 mM sodium phosphate pH 7.0), respectively. Substrates were 4 mg/ml O-nitrophenyl-β-D-galactopyranoside (ONPG (Sigma), molar extinction coefficient ε = 4.5 x 10^6^ M^-1^cm^-1^ at 420 nm) for the measurement of β-galactosidase activity and 4 mg/ml p-nitrophenyl-α-D-glucopyranoside (PNPG (Sigma), ε = 8800 M^-1^cm^-1^ at 415 nm [58]) for the measurement of β-glucuronidase activity. The kinetics of product formation over time were followed and the specific enzyme activities [mU/mg protein] were calculated by (ΔE/min x 10^6^) / (ε [M^-1^cm^-1^] x protein concentration [mg]).

### Electrophoretic mobility shift assay (EMSA)

Cells were glass bead-disrupted in TMEGA buffer (0.2 M Tris-HCl pH7.8, 0.3 M (NH_4_)_2_SO_4_, 10 mM MgCl_2_, 1.0 mM EDTA, 7.0 mM β-mercapto-ethanol, 10% glycerol). To each sample 1.0 mM proteinase inhibitor PMSF was added immediately before disruption. Supernatants were transferred to centrifuge cups for a Beckmann TLA 100.2 rotor and spun at 100,000 x g for 1 hour at 4°C in a Beckmann Ultima Max tabletop ultracentrifuge. The resulting S100 supernatants were retrieved and stored at -70°C in 20 aliquots. Protein concentrations were determined according to Bradford. Radioactive probes were prepared by filling in the *Eco*RI 3' recessed ends of a restriction fragment or double-stranded oligonucleotides with α-^32^P-dATP using Klenow DNA polymerase. The labeled fragments were purified by size exclusion chromatography on Sephadex G50 [67]. For assessment of protein-DNA interaction reactions (≤20 μl if possible) were set up in binding buffer (20 mM Hepes-NaOH pH7.8, 0.1 M NaCl, 10 mM MgCl_2_, 1.0 mM EDTA, 1.0 mM DTT, 10% glycerol, 0.2 mg/ml BSA) containing 10 fmoles of ^32^P- probe (≥2.0 x 10^4^ cpm) and 5.0 μg of sonicated calf thymus DNA to quench unspecific binding. Aliquots of the S100 extracts were thawed on ice and volumes corresponding to 10 to 40 μg of protein were immediately added to the reaction. Reactions were incubated 20 min. at room temperature and analyzed on a 4% polyacrylamide gel (acrylamide/bisacrylamide: 30/0.8) in TBE buffer (90 mM Tris, 90 mM H_3_BO_3_, 2.0 mM EDTA) that was run at 150 V for 2 to 2_1/2_ hours. The gel was transferred to Whatman paper and dried under vacuum at 80°C for 2 hours. Bands were visualized through autoradiography on X-ray film.

### Statistical analysis

Data were analyzed either by one-way ANOVA followed by Tukey’s post-hoc test or a Student *t*-test using GraphPad Prism version 3.02 for Windows, GraphPad Software, La Jolla California USA, www.graphpad.com.

## Supporting Information

S1 Fig Additional copy of *KlSIP4-6HA* suppresses *cat8*Δ and *snf1*Δ mutant phenotypes. (TIF)

S2 Fig Statistical analysis of differential expression in *S*. *cerevisiae* mutants. (TIF)

S3 Fig Statistical analysis of differential expression in *K*. *lactis* mutants. (TIF)

S4 Fig Binding of ScSip4-(HA)_6_ to ScCat8-regulated genes. (TIF)

S5 Fig Regulation of CSRE_*LAC4*_-complex by carbon source. (TIF)

S6 Fig (HA)_6_-tagged Sip4 and Cat8 variants are functional. (TIF)

S1 Table Yeast strains. (DOCX)

S2 Table Primer list. (DOCX)

## Abbreviations

CSRE: carbon source responsive element
*K*. *lactis*: *Kluyveromyces lactis*
*S*. *cerevisiae*: *Saccharomyces cerevisiae*
Sip4: SNF1 interacting protein 4
KlSip4: *K*. *lactis* Sip4
ScSip4: *S*. *cerevisiae* Sip4
Cat8: CATabolite repression 8
KlCat8: *K*. *lactis* Cat8
ScCat8: *S*. *cerevisiae* Cat8
Snf1: Sucrose non-fermentor 1 (α-subunit of heterotrimeric complex)
SNF1: Snf1-containing heterotrimeric complex
SEM: Standanrd deviation of the mean
EMSA: electrophoretc mobility shift assay
