# Supplementary figures and images for: Divergent Evolution of the Transcriptional Network Controlled by Snf1-Interacting Protein Sip4 in Budding Yeasts
Source: PLoS One. 2015 Oct 6;10(10):e0139464. doi: 10.1371/journal.pone.0139464 (PMC4634231; doi:10.1371/journal.pone.0139464)

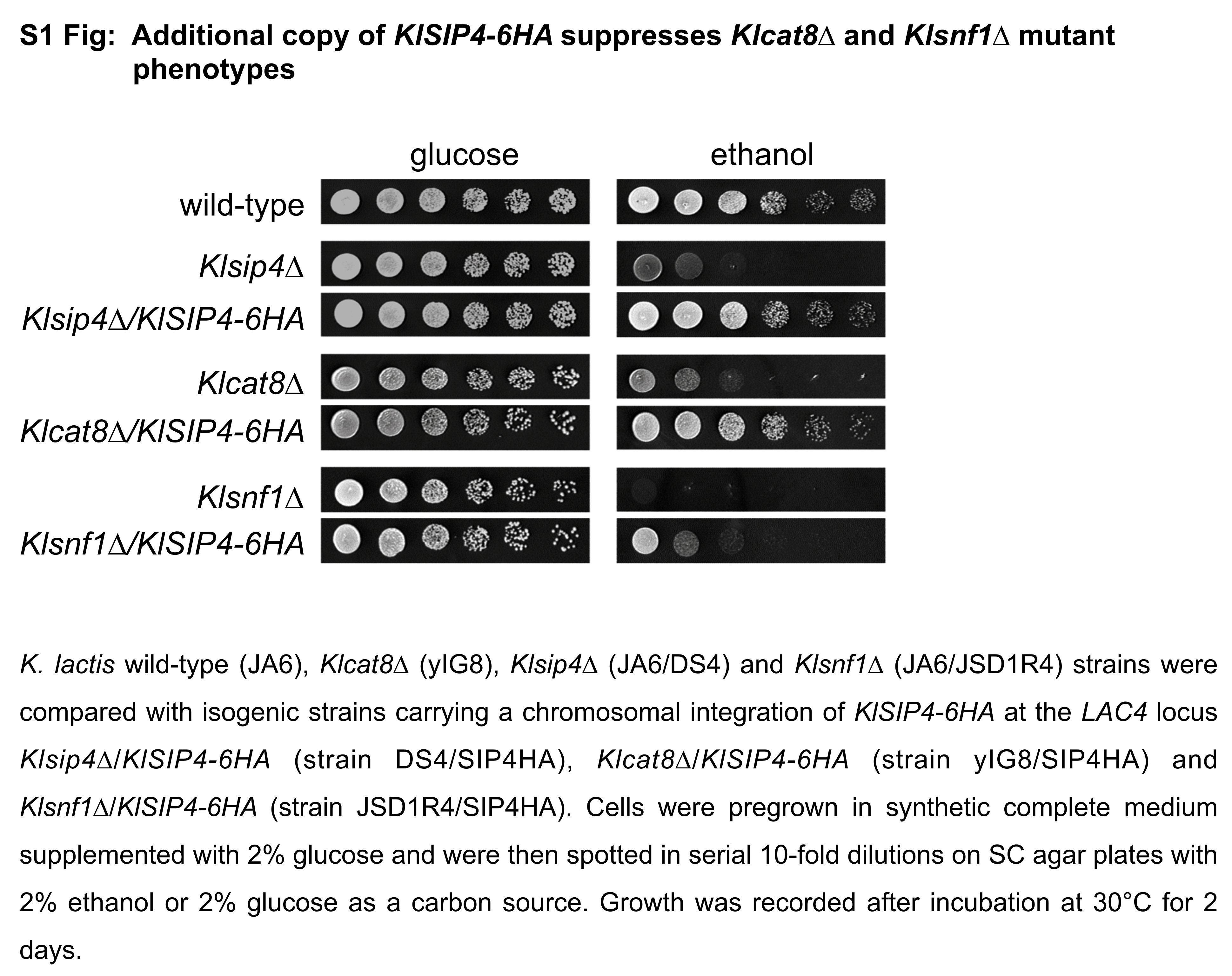

Supplement: S1 Fig — (TIF) [file pone.0139464.s001.tif]

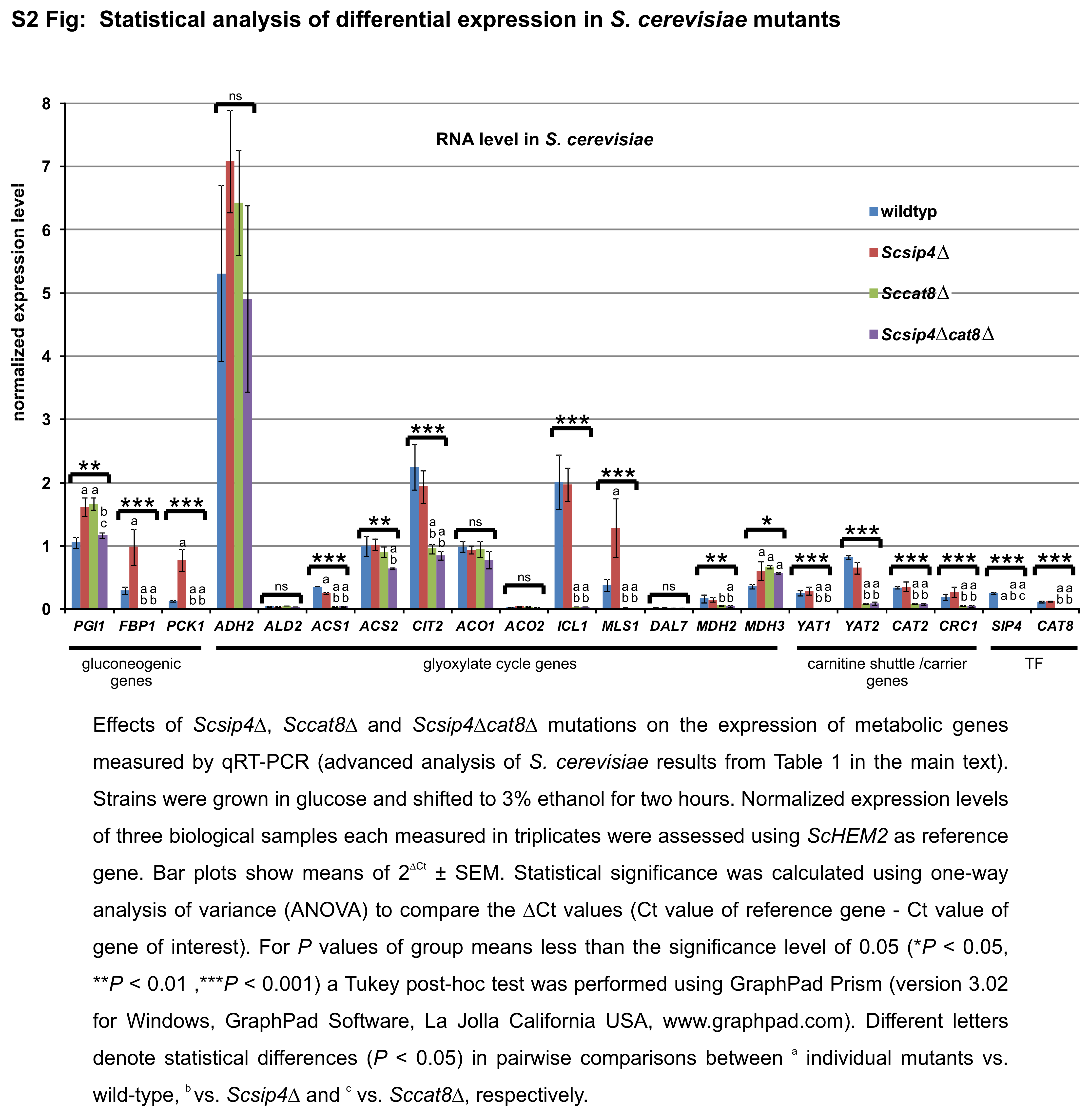

Supplement: S2 Fig — (TIF) [file pone.0139464.s002.tif]

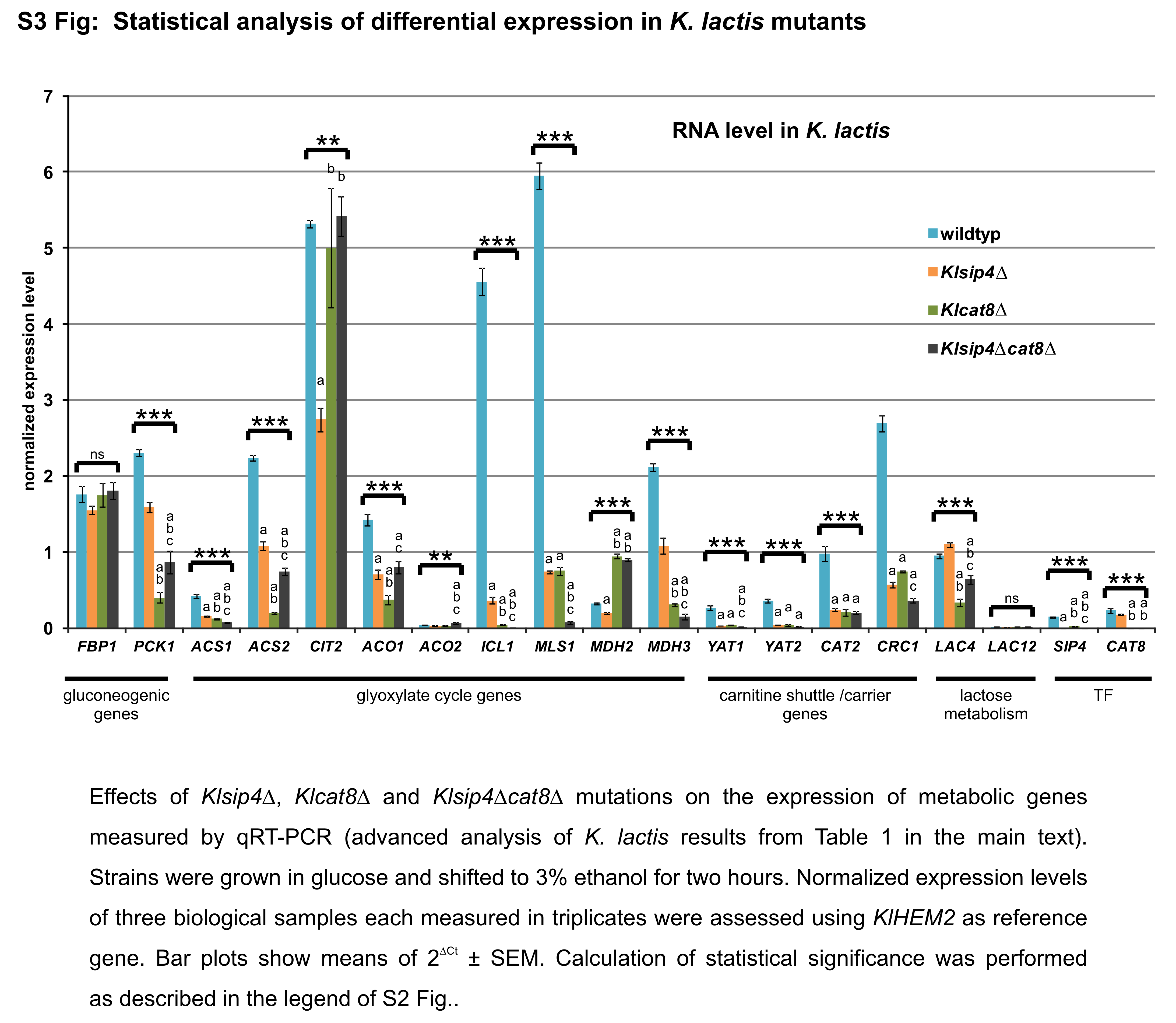

Supplement: S3 Fig — (TIF) [file pone.0139464.s003.tif]

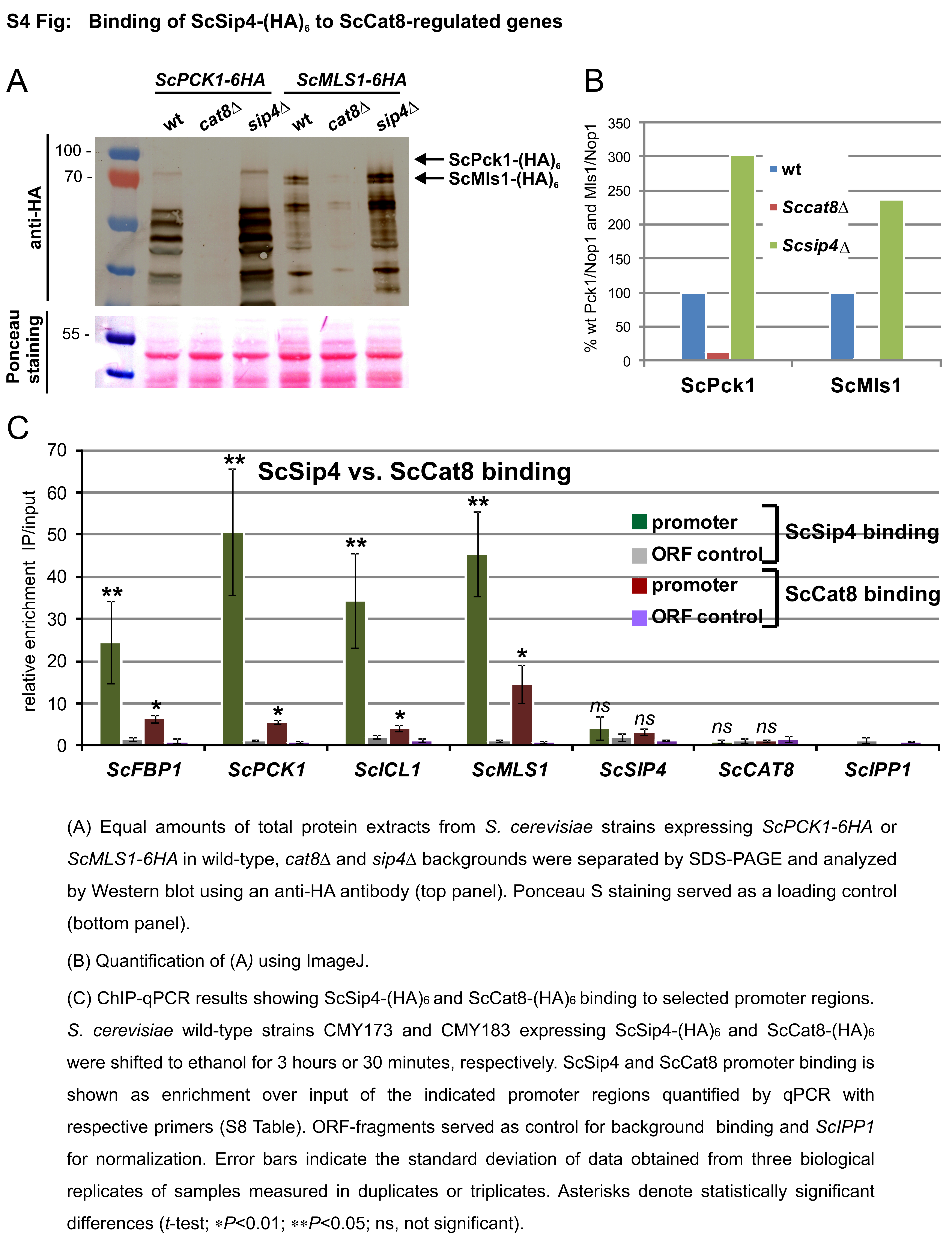

Supplement: S4 Fig — (TIF) [file pone.0139464.s004.tif]

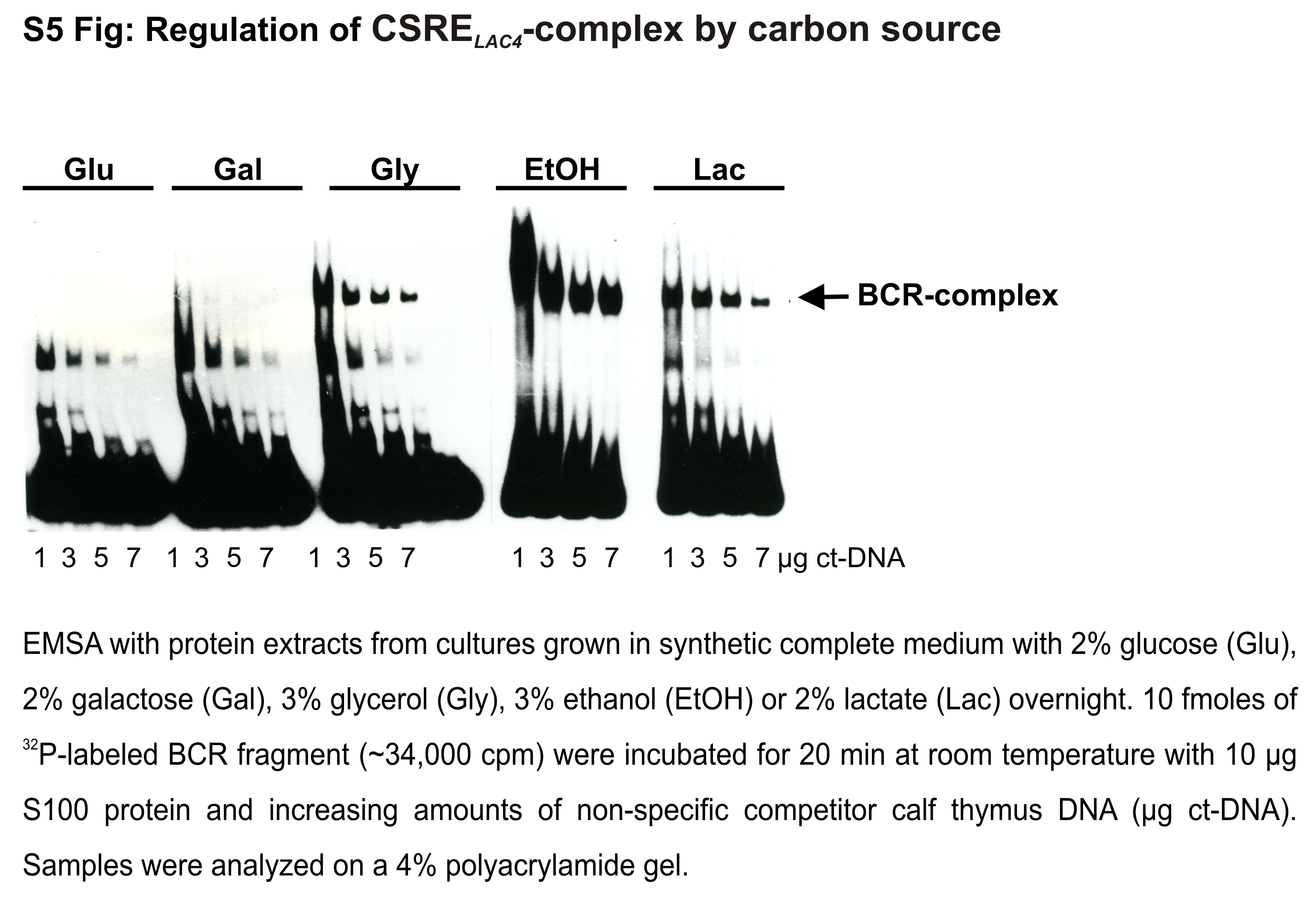

Supplement: S5 Fig — (TIF) [file pone.0139464.s005.tif]

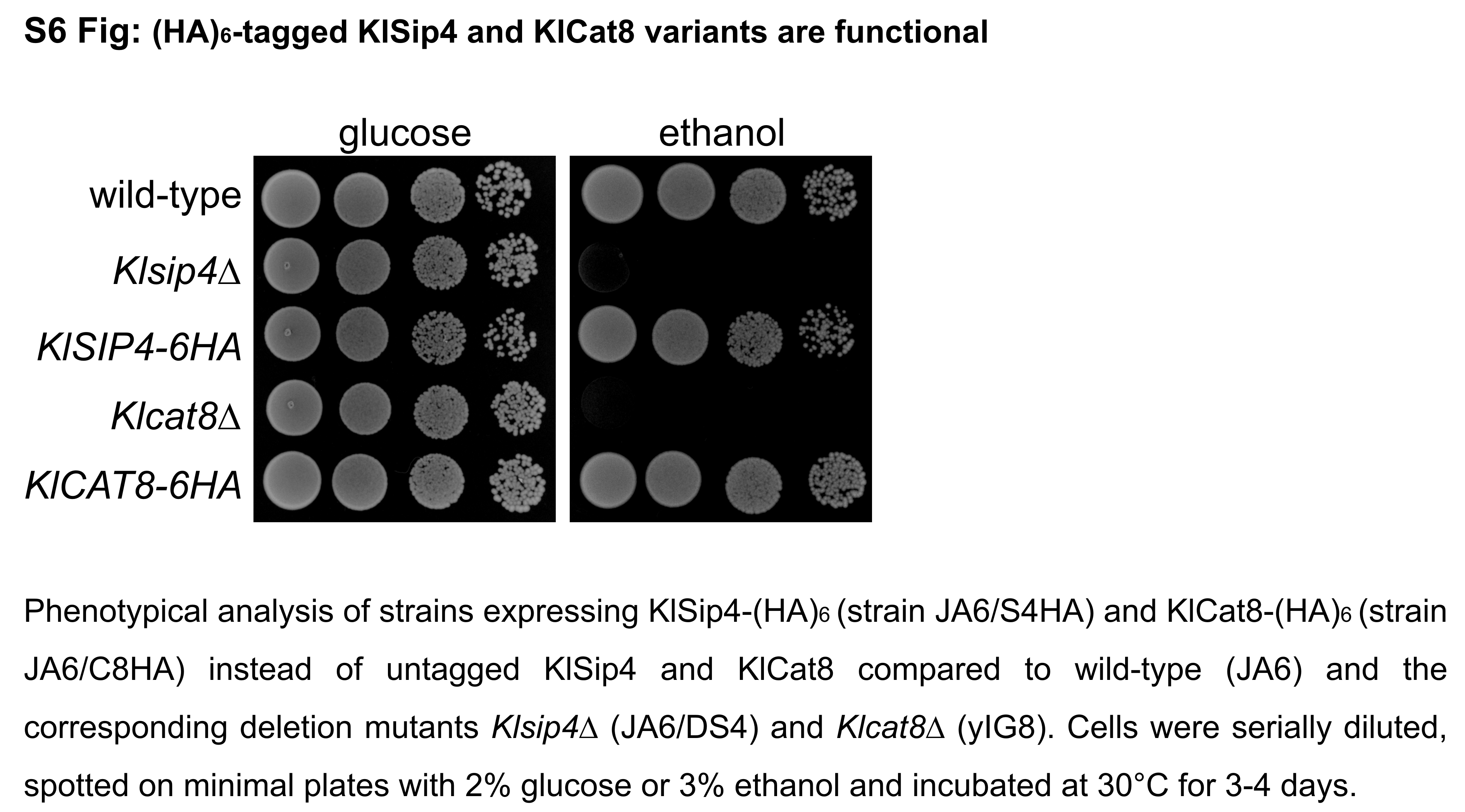

Supplement: S6 Fig — (TIF) [file pone.0139464.s006.tif]
