# Supplementary material for: Divergent Evolution of the Transcriptional Network Controlled by Snf1-Interacting Protein Sip4 in Budding Yeasts
Source: PLoS One. 2015 Oct 6;10(10):e0139464. doi: 10.1371/journal.pone.0139464 (PMC4634231; doi:10.1371/journal.pone.0139464)
Supplement: S1 Table — (DOCX) [file pone.0139464.s007.docx]

**S1 Table: Yeast strains**.

| Strain | Description | Source/Reference |
| --- | --- | --- |
| *K. lactis* |  |  |
| JA6 | *MAT*α *ade1*-*600 adeT*-*600 trp1*-*11 ura3*-*12 LAC9-2* | [1] |
| yIG8 | JA6 but *Klcat8*Δ | [2] |
| JA6/DS4 | JA6 but *Klsip4*Δ | this work |
| yIG8/DS4 | yIG8 but *Klsip4*Δ | this work |
| JA6/S4HA | JA6 but *KlSIP4-6HA-KlTRP1* | this work |
| JA6/C8HA | JA6 but *KlCAT8-6HA-KlTRP1* | this work |
| JSD1 | JA6 but *Klsnf1*Δ*::ScURA3* | [3] |
| JSD1R4 | JSD1 but *ura3*^−^ | [3] |
| JA6/LR2 | JA6 but deletion upstream of *LAC4* between -1068 and -1530 (ΔBCR) | [4] |
| JA6/LR2K | JA6 but replacing BCR by CSRE*_LAC4_* | [5] |
| JA6/SIP4HA | JA6 but *lac4::KlSIP4-6HA*-*KITRP1* | this work |
| DS4/SIP4HA | JA6/DS4 but *lac4::KlSIP4-6HA*-*KITRP1* | this work |
| yIG8/SIP4HA | yIG8 but *lac4::KlSIP4-6HA*-*KITRP1* | this work |
| JSD1R4/SIP4HA | JSD1R4 but *lac4::KlSIP4-6HA*-*KITRP1* | this work |
|  |  |  |
| *S. cerevisiae* |  |  |
| W303-1a | *MAT*a *ade2-1 his3-11, 15 leu2-3, 112 trp1-1 ura3-1 can1-100* | Laboratory stock |
| CMY187 | W303 but *Scsip4*Δ*::KlLEU2* | this work |
| CMY188 | W303 but *Sccat8*Δ*::KlLEU2* | this work |
| CMY189 | CMY188 but *Scsip4*Δ*::ScURA3* | this work |
| CMY196 | W303 but *ScYAT2-6HA-KlTRP1* | this work |
| CMY197 | CMY187 but *ScYAT2-6HA-KlTRP1* | this work |
| CMY198 | CMY188 but *ScYAT2-6HA-KlTRP1* | this work |
| CMY199 | W303 but *ScMLS1-6HA-KlTRP1* | this work |
| CMY201 | W303 but *ScPCK1-6HA-KlTRP1* | this work |
| CMY202 | CMY187 but *ScMLS1-6HA-KlTRP1* | this work |
| CMY204 | CMY187 but *ScPCK1-6HA-KlTRP1* | this work |
| CMY205 | CMY188 but *ScMLS1-6HA-KlTRP1* | this work |
| CMY207 | CMY188 but *ScPCK1-6HA-KlTRP1* | this work |
| CMY173 | W303 but *ScSIP4-6HA-KlTRP1* | this work |
| CMY183 | W303 but *ScCAT8-6HA-KlTRP1* | this work |

1. Breunig KD, Kuger P (1987) Functional homology between the yeast regulatory proteins GAL4 and LAC9: LAC9-mediated transcriptional activation in *Kluyveromyces lactis* involves protein binding to a regulatory sequence homologous to the GAL4 protein-binding site. Mol Cell Biol 7: 4400-4406.

2. Georis I, Krijger JJ, Breunig KD, Vandenhaute J (2000) Differences in regulation of yeast gluconeogenesis revealed by Cat8p- independent activation of *PCK1* and *FBP1* genes in *Kluyveromyces lactis*. Mol Gen Genet 264: 193-203.

3. Dong J, Dickson RC (1998) Glucose represses the lactose-galactose regulon in *Kluyveromyces lactis* through a *SNF1* and *MIG1*-dependent pathway that modulates galactokinase (*GAL1*) gene expression. Nucl Acids Res 25: 3657-3664.

4. Gödecke, A. (1990) Charakterisierung des LAC4 Promotors der Hefe Kluyveromyces lactis [dissertation].

5. Schmidt, T. (1995) Die Regulation der basalen Genexpression der Hefe-β-Galactosidase [dissertation]. Heinrich-Heine-Universität Düsseldorf.
