# Supplementary material for: Divergent Evolution of the Transcriptional Network Controlled by Snf1-Interacting Protein Sip4 in Budding Yeasts
Source: PLoS One. 2015 Oct 6;10(10):e0139464. doi: 10.1371/journal.pone.0139464 (PMC4634231; doi:10.1371/journal.pone.0139464)
Supplement: S2 Table — (DOCX) [file pone.0139464.s008.docx]

**S2 Table.** List of primer combinations used in this study.

| **Names primer pair** | **Forward sequence 5‘-3‘** | **Reverse sequence 5‘-3‘** |
| --- | --- | --- |
| **qRT-PCR in *K. lactis*** |  |  |
| KlYAT1-RT-F / KlYAT1-RT-R | AACTGGTACCTTGCCTACTG | GTTCAAAGAAGGTGGCAATC |
| KlYAT2-RT-F / KlYAT2-RT-R | GATGTGCTACCAAGAAATCC | TCAAGGCACTGACAAATCTC |
| KlCAT2-RT-F / KlCAT2-RT-R | AACTGGATGCAAGAACTATG | AGTTGGTAAAGGCTTATGAC |
| KlCRC1-RT-F / KlCRC1-RT-R | GGATGCTGCCAAACAGATAG | GGCAAAGTATAACGCACTTC |
| KlFBP1-RT-FW / KlFBP1-RT-RV | GATTGGCTGGTGCTTCAAAT | GCACCGTACATAGTGTAACA |
| KlPCK1-RT-F / KlPCK1-RT-R | TCACAATGTCCTCACCTTAC | TCACCGATCAACAGTCTATG |
| KlACS1-RT-F / KlACS1-RT-R | CAAGCGTATCGTCGATGAAG | CCAAATCCCTACCTGGAATG |
| KlACS2-RT-F / KlACS2-RT-R | AGATTGTCGACGAAGGTTTG | ATCTCTAGCTGGCTTCATTG |
| KlCIT2-RT-F / KlCIT2-RT-R | AGGCTTTGTCAGCAGATTTG | GTAACTGCCATCGAGAATTG |
| KlACO1-RT-F / KlACO1-RT-R | CCCAGGTGCTTTGATTATTG | CATGACATCGACAGCATCAG |
| KlACO2-RT-F / KlACO2-RT-R | GGGAATTGAAAGCTCCTAAG | TTAAGATACCAGCCAGTTTG |
| KlICL1-RT-F / KlICL1-RT-R | CTTTGGACCCTGTTCAAGTG | ACAGTGTCCATTGGGTAATC |
| KlMLS1-RT-F / KlMLS1-RT-R | CGGTCAAGTGAACTTGTATG | AGCCTCTTGGTCTAACAATC |
| KlMDH2-RT-F / KlMDH2-RT-R | AAGGCTAAGAACGGTAATGG | AATGTCGTCGATGGTATCTG |
| KlMDH3-RT-F / KlMDH3-RT-R | TTCTGGTGAGACCATCATTC | CAACTTCATCACCACCAAAC |
| KlSIP4-RT2-F / KlSIP4-RT2-R | CGGTGTACCGATTAATATCC | GGTTAGTACCGAGATCATTC |
| D01452(CAT8)F / D01452(CAT8)R | CAATCTTGGTGCAGGCATTG | ACTTGTGCCATCCTTCATCC |
| KlLAC4-RT-F / KlLAC4-RT-R | GAAGGACGGTAACATTACTG | AGAATCAAGTCCCTAACAAC |
| B14861G-F / B14861G-R (LAC12) | GTGTCTGGGTGTTGTTATTGG | CCACTTCTGCACAGTATGTTG |
| (ACT)-F / (ACT)-R | GCTCCAATGAACCCAAAGAAC | GTAACACCGTCACCAGAATCC |
| HEM2-F / HEM2-R | TTCAGAGATGCCGCTTGTTC | AGATTTCAGCTGCATCGGAC |
| ALG9-F / ALG9-R | ATACTGCGAGGGCGTTATTG | ACACTGGCATGAAACCATCC |
| IPP1-F / IPP1-R | TAAGGCTGTTGGTGACAACG | ATCGATGGCAATGACCTTCC |
| **qRT-PCR in *S. cerevisiae*** |  |  |
| ScYAT1-RT2-F / ScYAT1-RT2-R | AACTGGAGTGGAACCTCTAC | ACAGATAGTCCCTGATGTTG |
| ScYAT2-RT-F / ScYAT2-RT-R | GAACTTGTTCGGTACTACTC | TCCTCATCGCTTGAATAATC |
| ScCAT2-RT-F / ScCAT2-RT-R | AACTGTACTCTGTGGTATTC | ATAAGCTCCAGATGTACTTC |
| ScCRC1-RT-F / ScCRC1-RT-R | TTATCAGCGCTATTCCTACC | TGCCACCTTCTTTAACAATG |
| ScPGI1-RT-F / ScPGI1-RT-R | TTCCAACATTGACGGTACTC | TGATAGTTTCAGCGGTAGTG |
| ScFBP1-RT2-F / ScFBP1-RT2-R | CCTCTACGCATCTAGTATTG | GGCGGAATTCTTAAGTTAGG |
| ScPCK1-RT-F / ScPCK1-RT-R | AGTTCCCAGCCAATTTACAC | TTCATTTCACCGGCGTATTC |
| ScADH2-RT-F / ScADH2-RT-R | CTGGTATCACCGTATACAAG | AACAGCCAAAGAACCTAGAC |
| ScALD2-RT-F / ScALD2-RT-R | ATCAAACCTGCTGAGAATA | GCCTACAAGTGATCCATAAC |
| ScACS1-RT-F / ScACS1-RT-R | AGTGCTGACTTACTCTATGG | AAAGACTACGGAGTGAATG |
| ScACS2-RT-F / ScACS2-RT-R | ACTTACCTACCTCCTGTTTC | GCGGCACCTAATAAATAACC |
| ScCIT2-RT-F / ScCIT2-RT-R | GATTTCGTGGACTTGATGAG | AAGGGACAGATAAGGTGATG |
| ScACO1-RT-F / ScACO1-RT-R | CTGCCACTGCGAAATATAAC | TATGGGAGTCAGTACCAATG |
| ScACO2-RT-F / ScACO-2RT-R | AGGTGGTACTGGTTATATCG | AGGGAAAGTTGACGTTGTAG |
| ScICL1-RT-F / ScICL1-RT-R | AACTAGAGGCACGGTCAAAG | CACCACGCTCAATGAACATC |
| ScMLS1-RT-F / ScMLS1-RT-R | CGGTCAAGTTAATCTCTACG | GATAATAGTGGGCAAGTTGG |
| ScDAL7-RT-F / ScDAL7-RT-R | GATTTCGAGGACTCTTCATC | CTTCAACCTGTACTCCTTTC |
| ScMDH2-RT-F / ScMDH2-RT-R | GTCTTCGTTCTTGTCATTTC | ACACCCATGATCCTTCTTTC |
| ScMDH3-RT-F / ScMDH3-RT-R | AACTACAATGCACAGAAAGG | AAGTGCTCGTACTGCTTATC |
| ScSIP4-RT-F / ScSIP4-RT-R | AGCTCCTCTAACGCAATCAC | CCTCTCCTCGACAGTTTATC |
| ScCAT8-RT-F / ScCAT8-RT-R | CATCTTTGTGACGGCGTTTC | TTGAGTGCTACGTTGATGAG |
| ScHEM2-RT-F / ScHEM2-RT-R | TTCCGTGATGCTGCTTGTTC | ATAATACCGTCGGCACCTTC |
| ScRDN18-RT-F / ScRDN18-RT-R | GCGGCTTAATTTGACTCAAC | TCACTCCACCAACTAAGAAC |
| ScRPS26A-RT-F / ScRPS26A-RT-R | TCTACCCTGAATACGCTTTG | GTGGAGCTCTGTTCTTTCTG |
| ScIPP1-RT-F / ScIPP1-RF-R | GACCGATTGGAAAGTTATTG | TCTGAACCATTCGTTAGTAG |
| **knock out in *K. lactis*** |  |  |
| DSIP4-PR-5' / DSIP4-PR-TER | CGCGCGAATTCCGGATCCATCAATTAAGGG | GGATGTTGATTCTTCTGTTGGCGCTACTACAGG |
| DSIP4-PTREV / DSIP4-TER-3' | CCTGTAGTAGCGCCAACAGAAGAATCAACATCCG | AGCGGAATTCGAGCTCCCAATGCTAACCG |
| **knock out in *S. cerevisiae*** |  |  |
| Ko_Sc_SIP4_FW / Ko_Sc_SIP4_RV | AAAAAAAAAGTATATAAGGCAGAAAGCTTTCTGCCTCTCCGATTTCCTCTCCTACGGCCAGTGAATTCCCGG | TGCAAAAGGTTTAGCTGGGTATTGACCTTAGACGCGAAATTCGACTTGACTAGCTTGGCTGCAGGTCGACGG |
| Ko_Sc_CAT8_FW / Ko_Sc_CAT8_RV | AAACAGCCGTAAAATAGTAGATTTTGTTAAACTCCCCTTTAAACCTGTGAACGGCCAGTGAATTCCCGG | CCTTTGGTATATTCCAAAGAACCTCTAGCACAGAGCAAAGAAAATCTGAAGCTTGGCTGCAGGTCGACGG |
|  |  |  |
| **GUS reporter construct** |  |  |
| SIP4-P-for / SIP4-P-rev | AGGCTGCAGTTGAGCTCAACCTGTCTTTTTTCGAACT | CGTCTCCGACACCCGGGAGTGATATATACATATACT T |
| **6HA epitope tagging in *K. lactis*** |  |  |
| KlYAT2-IF1_FW / KlYAT2-IF1_RV | GGCGCCATCTCCTTGCATGCTTCCATTCAAGACATCCCAAACCTTTTGAAC | GTCGACCTGCAGCGTACGACCACGAGTATTAACTTTTCTACCAACACT |
| KlYAT2-IF3_FW / KlYAT2-IF3_RV | CGAGCTCGAATTCATCGATTTTATTCGGAGACATACCATACTCCCC | TATAGGGCGAATTGGAGCTCGAAGATACGGAGGTCGTTGCAG |
| TRP-IF2_FW / TRP-IF2_RV | CGTACGCTGCAGGTCGACTCCGGT | ATCGATGAATTCGAGCTCGCCTCGA |
| S3-KlSIP4 / S2-KlSIP4 | CTGATTTTTCACTTTTCAACAATAAGATCGACCACATCATAATCAAGGAGCGTACGCTGCAGGTCGAC | GATATTGCGGATGTTGATTCTTCTGTCTGAATTTTTCTGTTTGATTTGAGATCGATGAATTCGAGCTCG |
| S3-KlCAT8 / S2-KlCAT8 | CTGCTGGTGATTCATCAGTGTCCGATTTGCTGCGCTGGCAAAATGGAAATCGTACGCTGCAGGTCGAC | TAATGATAGTATGAAGTTTGTCCTGATGTCCTTCGGAAGCCTGGCTACTTATCGATGAATTCGAGCTCG |
| **6HA epitope tagging in *S. cerevisiae*** |  |  |
| S3-ScYAT2 / S2-ScYAT2 | TTGATTTTGACCGCAGTCGTGTGGGTAGAAAGGTGGCGACCTTAGATCAACGTACGCTGCAGGTCGAC | GCCAAGCTTACGCATAATGCTAGTAATAAATAGATAAACAAAGAGCGTTCATCGATGAATTCGAGCTCG |
| S3-ScSIP4 / S2-ScSIP4 | ACTCCAGTAAACAAAAATTCAAGATCCAGAATATTTTGAACTCGACCTTCCGTACGCTGCAGGTCGAC | TCACTGCTATCAGAGGAAGAGCTGAATGAGTAATACGAAGGAGCGTAATGATCGATGAATTCGAGCTCG |
| S3-ScCAT8 / S2-ScCAT8 | GTTCCAACACAGATAATGTATCTGATTTATTCCAATGGCAAAACGCCAAACGTACGCTGCAGGTCGAC | GAATATTTAGAGGATTCCGTTTTGAATATATTACACTATGAAATAAAGAAATCGATGAATTCGAGCTCG |
| S3-ScPCK1 / S2-ScPCK1 | ATCAAGACAGAGCCACACCAGATGTATTAGCCGCTGGTCCTCAATTCGAGCGTACGCTGCAGGTCGAC | AATTCTTTTTTTTTTTTTTGGATTGAACATATCGAACGAACATGTTTCGTATCGATGAATTCGAGCTCG |
| S3-ScMLS1 / S2-ScMLS1 | ACGACGAAATTGTGTCCACTAAGGCGACGCCCACTGATTTGAGCAAATTGCGTACGCTGCAGGTCGAC | ATATAGGCATGAATATATTTTTATATATGTGTACACTGGGGCAAGGGAGAATCGATGAATTCGAGCTCG |
|  |  |  |
|  |  |  |
| **ChIP-qPCR in *K. lactis*** |  |  |
| KlMLS1-CSRE-F / KlMLS1-CSRE-R | CGTTACCCGAATACAAAGAC | AAACTCACGCAATCAGTATG |
| KlICL1-CSRE-F / KlICL1-CSRE-R | AAGGACGAATGACACAAGAC | TAACCTGCTATTTGGCTGAG |
| KlSIP4(DPB11)-CSRE-F / KlSIP4(DPB11)-CSRE-R | TTTGCAATTGTCCCTTTCAG | ATTCGGACTTGTGACTTCTC |
| KlCAT8-CSRE-F / KlCAT8-CSRE-R | GTTCATCGTATCCACTACTG | CTCGACCATCGAAATAAATC |
| KlYAT2-CSRE-F / KlYAT2-CSRE-R | ACACCAACCACCATTAATCC | GCCTAGAAAGAGATGTAGAG |
| LAC4-(-1000)fw / LAC4-(-1000)rv | TCCTTTGGCCTTCCAAGTCG | AAGCCAGTCCACAGTCCATC |
| **ChIP-qPCR in *S. cerevisiae*** |  |  |
| ScFBP1-CSRE-F / ScFBP1-CSRE-R | ATAGACAACCCATCAAACTG | TCTTACGCCCTTAACATTAC |
| ScPCK1-CSRE1-F / ScPCK1-CSRE1-R | CGATCCACCGGAGAATATCG | CCCGGAGCTTCAGTGATATG |
| ScMLS1-CSRE-F / ScMLS1-CSRE-R | TTCGTGCTTAGTGATGTCTC | TCTATCCGTCGACTAACTTC |
| ScICL1-SIP4BSfw / ScICL1-SIP4BSrw | TGTGGTGATTGGCTTCAGTT | CGGGTTTTGCTACTCGTCAT |
| ScSIP41-CSRE-F / ScSIP41-CSRE-R | GGTGTGGGTGCAATCAAGAC | AAGAGGATTAGCCGCTGAAC |
| ScCAT8-CSRE-F / ScCAT8-CSRE-R | AATAAGGAGGGCAATGCAAG | TGTATATGTGCGAATGTGTC |
